# Ambient, real-time digitization and datafication of glass slide microscopy towards AI-at-the-microscope

**DOI:** 10.1038/s41467-026-77887-1

**Published:** 2026-09-17

**Authors:** Cooper Maira, Max S. Cooper, Kimberly L. Ashman, Andrew B. Sholl, Sharon E. Fox, Shams Halat, David Manthey, Roni Choudhury, Jonathan Sears, Carola Wenk, J. Quincy Brown, Brian Summa

**Affiliations:** 1 Department of Computer Science, Tulane University, New Orleans, LA USA; 2 Department of Biomedical Engineering, Tulane University, New Orleans, LA USA; 3 Touro Infirmary, New Orleans, LA USA; 4 Southeast Louisiana Veterans Healthcare System, New Orleans, LA USA; 5 Department of Pathology and Laboratory Medicine, Tulane University, New Orleans, LA USA; 6 Kitware, Inc., Clifton Park, NY USA

**Keywords:** Biomedical engineering, Pathology, Applied optics, Applied mathematics

## Abstract

Pathology remains central to clinical diagnosis, yet adoption of digital pathology is constrained by financial, operational, and workflow burdens of fully digital infrastructure. We introduce HistoCAM, a platform for ambient, real-time datafication and digitization of glass-slide microscopy that preserves microscope workflows. A 31-megapixel, high space-bandwidth-time-product camera and custom software application stream and composite the pathologist’s eyepiece view, passively generating multi-resolution images from 2X to 40X while recording magnification use, search paths, and dwell times. These outputs provide immediate workflow uplift through digital annotation, measurement, quality assurance, and real-time integration of configurable AI tools. Simultaneously, HistoCAM links image content with expert interaction data and supports rapid generation of annotated, pre-embedded training data during routine slide review. By converting routine microscopy into an AI-ready data stream without requiring additional acquisition steps, HistoCAM provides a practical bridge to computational pathology while creating process-aware datasets that capture how pathologists examine and interpret tissue.

## Abstract (web summary)

HistoCAM ambiently captures the pathologist’s full, optically sampled microscope view, enabling real-time digital and AI tools while turning routine slide review into process-aware, AI-ready data.

## Introduction

Pathology is foundational to human health research and clinical practice, underpinning virtually every modern advance in diagnosis, prognosis, and precision medicine. For well over a century, clinical pathologists have relied on direct microscopic observation of glass slides as the gold-standard workflow. During that time, there have been major advances in imaging systems and computational algorithms, and since 2017, several whole-slide imaging (WSI) scanners have obtained FDA clearance for primary diagnosis on digitally-scanned slides^1^. Digital slides enable telepathology and AI assistance^2–7^, and are created by robotic automated microscopes which digitize glass slides at high resolution (typically at 20X to 40X magnification^8^, or 0.5 to 0.25 μm per pixel, respectively). However, although WSI has been increasingly adopted for research and training purposes, widespread clinical impact has yet to materialize, since fewer than about 10% of clinical laboratories have replaced (or intend to replace) routine microscopy with WSI^9^. The chief barriers are cost and implementation complexity, given that a single WSI system can exceed the price of an entire microscope fleet for a typical pathology department, and requires hiring dedicated personnel for scanning, quality control, and data management. Moreover, since digital pathology does not eliminate the need for slide preparation, slide scanning represents an additional step in the traditional sign-out workflow. For this reason, many hospital leaders remain hesitant to disrupt well-established and relatively inexpensive microscope-based workflows without a clear and immediate return on investment.

Because the vast majority of slides generated in clinical practice are never digitized, the reach of emerging FDA-approved computational pathology tools^10^ in routine care to pathologists and patients remains limited. However, the recent interest in AI applications to digital pathology represents a driving factor for digital pathology adoption, if the cost and implementation barriers to digitization can be overcome. Several groups have proposed bridging solutions to ease digitization constraints. The Augmented-Reality Microscope (ARM)^11^ for example, superimposes AI overlays in the eyepieces, but both the algorithm and the user are confined to a single field-of-view (FOV) at a time. Without a digital representation of areas larger than a single eyepiece FOV, the algorithm and the pathologist are limited to the context provided in a single FOV, which, even at low magnification, may not contain the entire specimen area or region of interest, and furthermore does not retain any memory of other areas observed by the pathologist that contributed to the diagnosis. Video-mosaicking platforms that stitch slides via automated stages^12–19^ or manual sweeping^20,21^ improve coverage in a single magnification^22–25^, yet primarily because these solutions use camera sensors that underfill the full microscope FOV, they require the pathologist to look away from the eyepieces to ensure gap-free coverage and regulate stage speed using a computer monitor – all of which are alterations to established behaviors and workflows that slow diagnosis. To our knowledge, no system passively converts the entire diagnostic session at the microscope into a multi-resolution digital slide while preserving the natural glass slide diagnostic workflow, capturing the pathologist’s full visual experience and search behavior.

We therefore sought an ambient approach that digitizes everything the pathologist actually sees during the diagnostic process. We define ambient digitization as the passive, unobtrusive capture of the pathologist’s eyepiece view in real-time, producing variable-resolution slide images and search telemetry without requiring workflow changes or additional user effort. Here, we introduce the Histopathology Computer Assisted Microscope (HistoCAM), a platform that automatically digitizes every region a pathologist inspects, across all objective lenses, in real time while preserving a full spatiotemporal history of the visual analysis. HistoCAM couples a large-FOV, high space-time-bandwidth camera to a conventional brightfield microscope via a commercial trinocular beamsplitter that simultaneously samples the entire eyepiece FOV, and custom software that: (1) streams 31 megapixel (MP) video at frame rates sufficient to prevent motion blur during normal slide movement, (2) detects objective changes automatically and spawns scale-aware connected components to create a multi-resolution slide image (MRSI) spanning 2X to 40X magnification, (3) logs magnification-specific dwell times and search paths, generating a rich behavioral telemetry layer unavailable in traditional WSI, and (4) vector-embeds and feeds tiles to user-configured AI models on-the-fly, enabling real-time connection to digital AI support without delaying sign-out. Critically, the pathologist remains at the eyepieces; HistoCAM runs unobtrusively in the background, introducing no additional steps and limited additional hardware beyond a camera and computer. The resulting MRSI’s are fully compatible with existing digital pathology tools for annotation, measurement, and archiving, while the behavioral logs open new avenues for training models that emulate expert reasoning, as well as enabling immediate quality-assurance checks during diagnosis, such as did-I-see-everything coverage checks, or dwell-time saliency alerts.

**Fig. 1:**
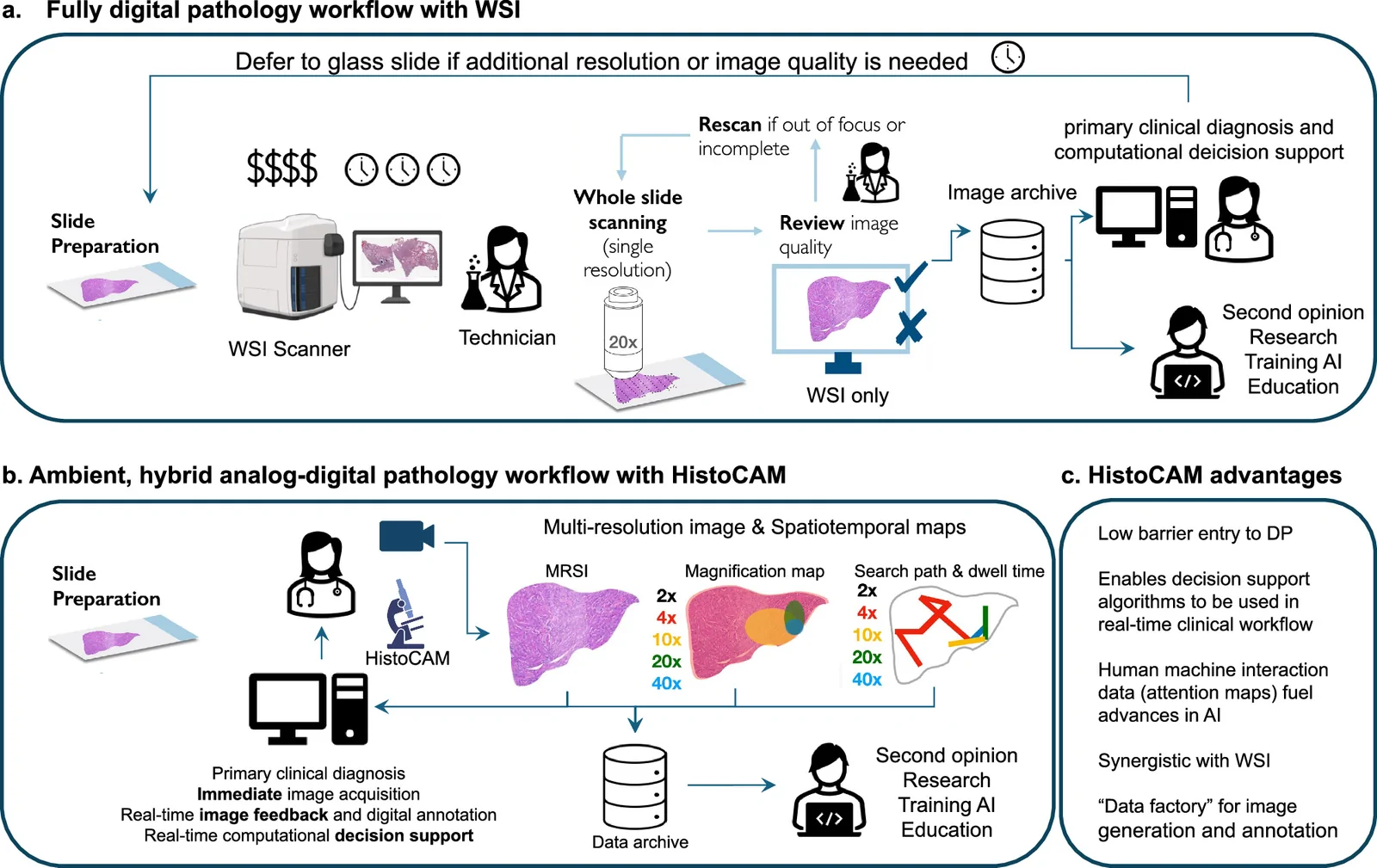
Conceptual comparison of fully digital WSI workflow with the real-time, ambient, hybrid analog-digital workflow of HistoCAM. **a** Fully digital pathology workflow with a Whole Slide Imaging (WSI) system. **b** Ambient, hybrid analog-digital pathology workflow with Histopathology Computer Assisted Microscope (HistoCAM) to create multi-resolution slide images (MRSI). Red shading and lines represent use of the 4X objective lens, yellow for the 10X, green for the 20X, and blue for the 40X. **c** Advantages of the HistoCAM digitization and datafication platform relative to traditional WSI scanning. Created using BioRender. Brown, Q. (2026) [https://BioRender.com/oyreipw].

By converting the current glass slide workflow itself into an inherently digital and AI-ready data stream, HistoCAM addresses the cost and workflow barriers that limit traditional WSI adoption and provides a practical path for computational pathology deployment at the ROI and whole slide level, even in laboratories that cannot justify a fully digital workflow using WSI scanners. Importantly, it provides a platform that could promote more widespread digitization and improve the current clinical practice of pathology using glass slides, while laying the foundation for future, process-aware advances in computational pathology (Fig. 1).

## Results

### Design and development of HistoCAM

**Fig. 2:**
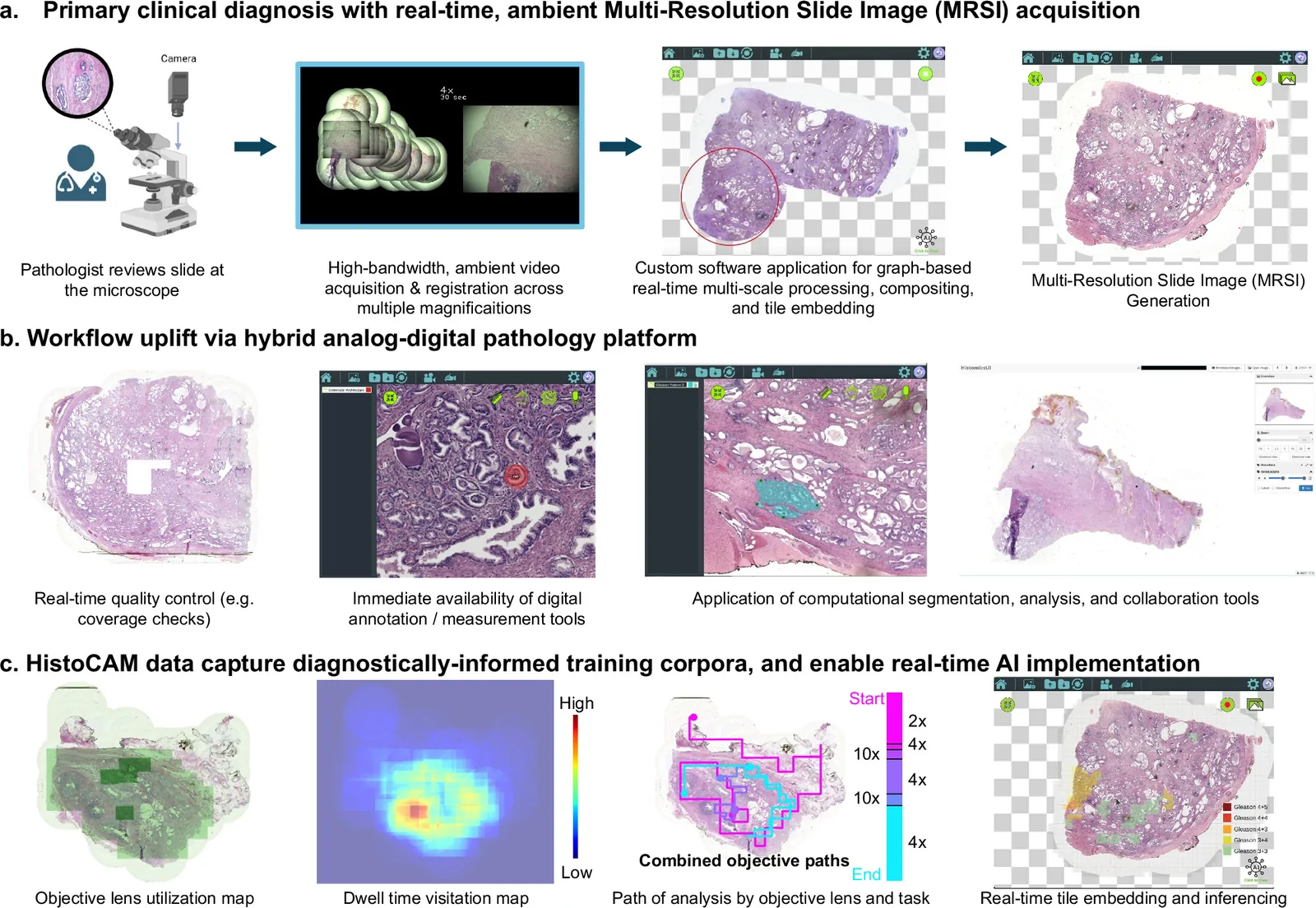
Overview of the HistoCAM platform. **a** The pathologist operates the microscope according to current established standards for glass slide analysis, viewing the slide through the eyepieces. Simultaneously, a trinocular beamsplitter mirrors the full eyepiece FOV onto a high bandwidth camera which continuously streams video at ~26 fps. A custom software application is used for efficient frame registration, processing, compositing, and tile-embedding, resulting in a Multi-Resolution Slide Image (MRSI) immediately at the conclusion of glass slide review. **b** Real-time use of HistoCAM enables a workflow uplift from a fully analog workflow, providing digital quality control, annotation and measurement, and application of computational segmentation, analysis, and collaboration tools. **c** By allowing pathologists to view the slide through the eyepieces 100% of the time during digitization, the resulting data reflect information about the processes contributing to the clinical diagnosis, such as magnification, dwell time, and search path maps. MRSI’s are tiled and feature-embedded in real-time, enabling a data factory for rapid generation of training corpora for pathologist-informed AI models, and real-time implementation of such models. Objective lens utilization is shown with varying shades of green; low-power objectives are lower opacity, and higher-power objectives are more opaque. Dwell time visitation is represented with red in high dwell areas, with a gradient to blue in low attention areas. The path of analysis begins with magenta (2X), transitions to purple (4X and 10X), and ends with cyan (4X). Created using BioRender. Brown, Q. (2026) [https://BioRender.com/ljtgiqx].

We built HistoCAM, a system that bridges the gap from the gold standard of analog clinical pathology to the rapidly growing innovations in digital pathology (Fig. 2). HistoCAM introduces digital capabilities into the existing clinical glass slide workflow without altering the pathologist’s behavior. As a pathologist-driven tool, HistoCAM generates multi-scale mosaics from glass slide reviews using the traditional brightfield desktop microscope without specialized stage automation hardware. The result of employing HistoCAM during glass slide review is a digitized, multi-resolution whole slide image (MRSI) from the recording of unconstrained slide review under the microscope. A detailed comparison of HistoCAM with prior microscope-based digitization and augmentation systems, highlighting differences in field-of-view and optical resolution coverage, workflow integration, multi-resolution behavior, and telemetry capture, is provided in Supplementary Table 1 and Supplementary Note 1. In contrast to these competing methods, we ambiently generated digital images of the entire specimen that were directly reflective of natural glass slide diagnostic processes by leveraging a high space-bandwidth-time-product camera capable of fully digitizing the optical information available to the pathologist through the eyepieces, and a computationally efficient processing approach. These distinctions reflect a shift from single-frame-based (snapshot) or manually-mediated mosaic digitization approaches toward an ambient, task-driven paradigm aligned with natural diagnostic behavior.

To achieve this, we first determined the engineering constraints and specifications required to successfully capture unconstrained, full-FOV slide review by pathologists. Our design requirements were: (1) the system should enable simultaneous splitting of the slide image to the eyepieces and the camera; (2) the camera should cover all, or most, of the available microscope field of view (FOV) across all magnifications, without resorting to a demagnification adapter which matches the FOV to the camera size at the expense of resolution; (3) the camera frame rate should be high enough to provide images free of motion blur during natural and unconstrained slide movement by the pathologist; and, (4) the pixel size of the camera should be small enough to provide adequate sampling of the available optical resolution for every objective lens mounted on the microscope. To determine the speed at which a pathologist moves a slide for each magnification while looking exclusively through the eyepieces, we built the first HistoCAM prototype (HistoCAM v0) using a camera (FLIR Blackfly S) with a small FOV, but a high frame rate, and used it to record videos of a pathologist reviewing a slide while looking solely through the eyepieces across different magnification lenses at 250 frames per second (fps). By applying a pairwise registration to each successive frame in the stream, we were able to measure the translation distance. By also recording the time increment between each frame, this allowed us to calculate the instantaneous translation speed over the duration of the viewing sessions, as a function of objective lens magnification (Supplementary Fig. 1). We found that the maximum translation speed varied depending on the level of magnification used, as expected, and ranged from 7 mm/s at maximum and 1.8 mm/s on average for the 4X objective lens, to 2 mm/s maximum and 0.3 mm/s on average for the 20X objective lens. We used these data to construct design curves that related the minimum frame rate as a function of sensor width for different objective magnifications, to ensure at least 20% overlap between adjacent frames. These experiments also confirmed that if the sensor underfills the eyepiece FOV, gaps in slide coverage will result when pathologists look solely through the eyepieces during analysis and digitization (Supplementary Fig. 8, Supplementary Movie 1).

Next, to satisfy the requirement that the camera sample the entire eyepiece FOV with adequate sampling density to ensure alias-free reconstruction of the available optical resolution, we determined that for our microscope fitted with 22 mm FOV eyepieces, the ideal camera sensor has a diagonal of at least 22 mm, with a space-bandwidth product (SBP) of 32 megapixels, based on the maximum space-bandwidth product of typical objective lenses used in clinical pathology (generally, the lowest magnification objective lenses have the highest SBP). We chose to use Nikon microscopes with 80%/20% (camera/eyepiece) trinocular beamsplitter heads as a platform. Combining these requirements, we then conducted a search for an appropriate camera sensor. We settled on a camera with a Sony IMX342 sensor (FLIR Oryx Color 31MP 10 GigE), which is a 31.36 megapixel (MP), 4:3 APS-C sensor format, with 6464 × 4852 pixels, 3.45 μm pixel size, a diagonal of 27.9 mm, and a frame rate of 26 frames per second (fps), resulting in a space-bandwidth-time-product (SBTP) of ~815 megapixels/s. This sensor provides complete to near-complete coverage of the eyepiece FOV and available optical SBP for all objective lenses tested (92% to 99% FOV coverage; 98% to >100% SBP coverage) (Supplementary Table 2), and near video-rate speed exceeding the minimum space-bandwidth-time requirement to avoid gaps in coverage at the highest expected stage translation speed. We proceeded with this sensor for the next stage of HistoCAM development (HistoCAM v1).

### Generating an efficient low-power whole slide preview in real-time

**Fig. 3:**
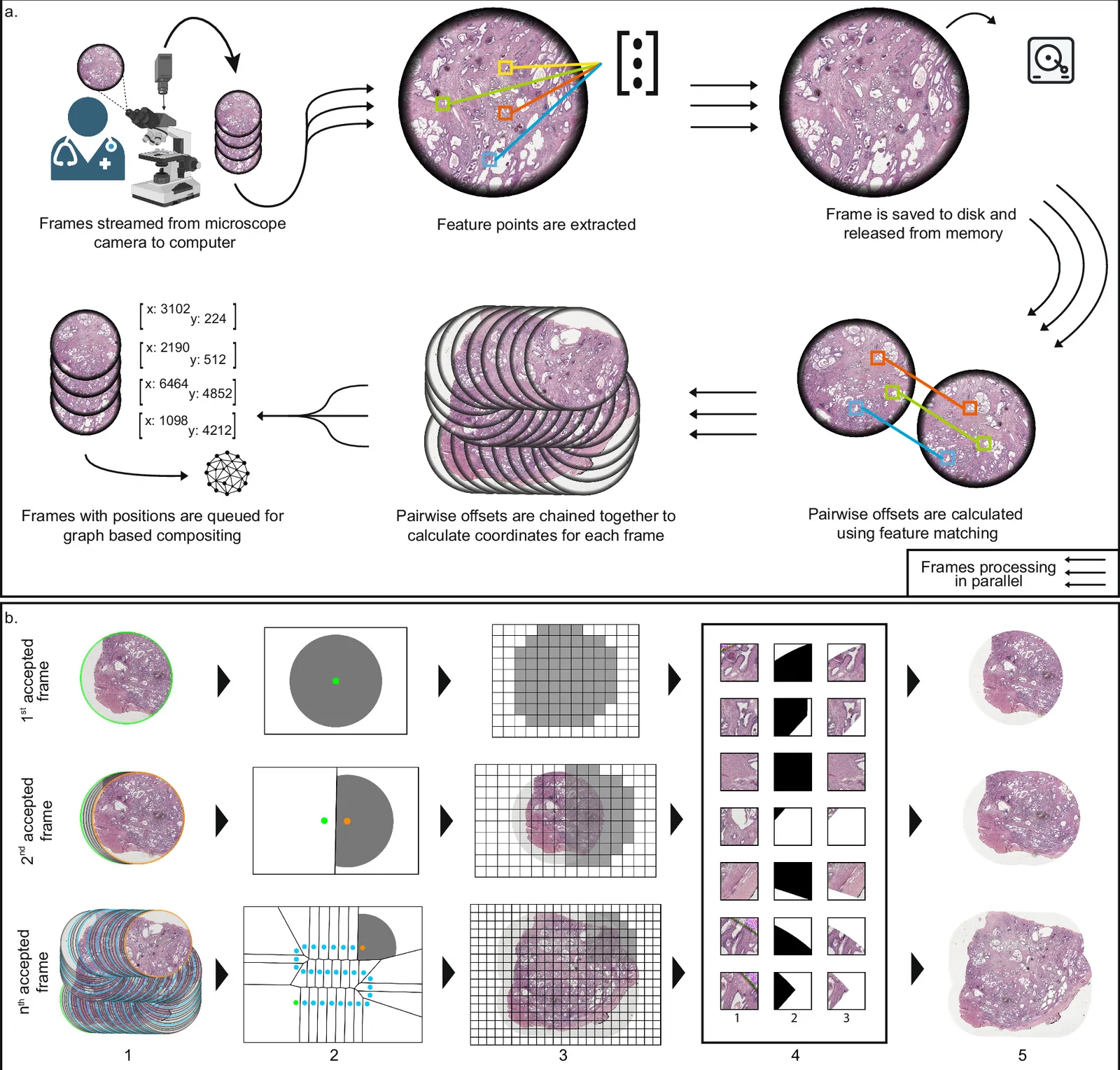
Real-time graph-based compositing from unconstrained slide review for a 2X coverage preview. **a** Top Panel: Frames are streamed from the camera covering pathologist analysis to the computer in Raw format. Frames are subsequently debayered, filtered for quality, and flatfield corrected. Feature points (yellow, green, red, and blue) are then extracted from the frames, and frames are saved to disk and released from memory. Pairwise offsets are calculated using feature matching between the extracted key points from each frame (red, green, blue), and are then chained together sequentially to calculate absolute coordinates for each frame. Every frame with a position is then queued for graph-based compositing. **b** Bottom Panel: Shows the process of adding data from registered frames in a continuous stream. (1) The initial frame is accepted (green). Frames are rejected until a minimum pixel distance has been traversed (grey). Subsequent accepted frames are shown in blue, with the most recently accepted frame in orange. (2) A newly accepted frame is added to the Voronoi graph, then the frame’s Voronoi face is intersected with the frame’s boundary leaving the pixels that the frame will contribute (i.e., the copy-mask, shown in grey). If the copy-mask is less than 20% of the frame area, the frame is rejected and removed from the Voronoi graph. (3) Projecting the copy-mask onto the composite grid gives the affected tiles. (4) Each affected tile is submitted as a separate copy task. (4.1) is frame data at the grid tile ROI; (4.2) is the copy mask at the grid tile ROI; (4.3) is data to be copied from the frame to tile. (5) Tiles are rendered after update. Created using BioRender. Brown, Q. (2026) [https://BioRender.com/3q6ed1c].

For the first demonstration of real-time utility with HistoCAM v1, we aimed to generate a low-magnification whole slide preview of the recording session in real-time. The driving idea behind the low-magnification whole-slide preview, gleaned from previous recording sessions with a pathologist that were mosaicked in post-processing using the HistoCAM v0 prototype and registered to standard WSI’s of the same slides, was that most of the time, but not always, pathologists covered the entirety of the slide with low-power objectives (Supplementary Figs. 7, 9, 10); and that higher-power observations typically resided completely within the areas covered by the lower-power objective lenses. We reasoned that, by first generating a low-magnification whole-slide preview, we could alert pathologists to regions not examined during the diagnostic search. A key aspect of the utility of this approach is the ability to deliver such a coverage preview to users in real-time. To achieve this, we developed a custom acquisition software application in C++ (HistoCAM App, Supplementary Fig. 2) which we used to efficiently control recording of the image streams and to process the data as it came off the camera. We sampled the data stream for only those frames collected with the 2X objective lens and then used a compositing approach to reconstruct this low-power mosaic in real-time (Fig. 3). Specifically, as a pathologist navigated the slide, the incoming sequence of frames was feature-extracted using Oriented FAST and Rotated BRIEF^26^ (ORB), quality-assured (exposure levels, focus quality), and spatially registered. We opted to use ORB for single-resolution feature matching as it is computationally more efficient than the Scale Invariant Feature Transform SIFT^27^ while offering similarly accurate feature extraction and matching across images of the same magnification level. Each of these tasks was performed across multiple threads in our parallelized system. As frames arrive at their absolute coordinates, they leave the parallel system and are sequentially evaluated by our graph-based compositing approach.

The first challenge was that, at the high SBTP of 815 megapixels/s, a large volume of data had to be processed in real time. Specifically, with this sensor, up to 48 GB of data is generated per minute of continuous recording time, with slide review times for a single slide varying from <1 to several minutes. To composite a mosaic from such high-volume video streams, it became imperative to prioritize efficient sampling of the image frames to ensure complete coverage while minimizing processing and storage of redundant frames. Our initial, naive approach for sub-sampling the data stream was to reduce the number of frames using simple decimation. Depending on the computed overlap, every 5th or 10th frame would be kept for the mosaic, and the rest would be discarded. Although this method was often successful in initial tests, it posed a risk of coverage gaps from variability in the pathologist’s translation speed and lacked data efficiency; therefore, it was not pursued further.

Instead, we developed a graph-based sampling and compositing approach. First, we note that this frame selection problem reduces to a min-set coverage problem, which is known to be NP-hard. Therefore, our solution is approximate and must balance accuracy with computational speed. We therefore implemented a computationally efficient, graph-based approach that assumes pixel quality decreases with distance from the frame center due to angle of capture and potential imperfections in the lens^28^. Once a frame’s absolute coordinates have been determined, it is added as a site in a Voronoi graph. A Voronoi graph is a geometric structure that partitions a space into regions around a set of sites, where each region contains all the locations closer to its defining site than to any other. Adding a single site to a Voronoi graph has worst-case complexity *O(n)*, where *n* is the number of existing sites in the graph. For most slide composites, the number of existing sites does not exceed 100, and this expense is trivial in the context of image processing. The area of the resulting Voronoi face is then computed. This face is considered to be the region of the final composite where the new frame contributes higher-quality pixels than any other frame. If this area is less than 20% of the frame’s pixel area, the frame is rejected and removed from the Voronoi graph. If the area contribution is sufficient, the frame is added to the composite and features are extracted using the SIFT^27^ algorithm for later alignment and potential frame matching across objective lens (i.e., scale) changes.

Note that this process is robust to pauses in the slide movement. For instance, if the user leaves the microscope and the slide remains static, the camera continues to acquire frames without translation. The corresponding Voronoi sites then overlap, producing small faces that are insufficient for inclusion in the composite. Frames collected during these periods are, as a result, discarded, and there is no risk of data pileup. This ensures that natural viewing behavior is preserved, without pressuring the user to monitor or time-limit the session. By utilizing parallel processing and requiring a sufficient contribution during graph-based compositing, HistoCAM whole slide previews were efficiently sampled and reconstructed in order to provide a real-time did-I-see-everything slide coverage preview of the 2X magnification lens only.

### Multi-resolution slide images generated in real-time

Achieving our goal of whole slide image mosaic previews for the low-power objectives in real-time was a useful step in HistoCAM development and utility for end-users. However, our goal was to reconstruct the complete history of slide review across all objective magnifications, thereby capturing all visual information available to the pathologist during gold-standard clinical review. To that end, HistoCAM had to generate images in real-time that reflected the hierarchical process by which pathologists identify and confirm areas of diagnostic interest across multiple objective lenses. Having previously demonstrated the ability to efficiently composite at a single resolution, we needed a method for automatically detecting and performing scale-aware registrations between each magnification level as the pathologist switched between objective lenses.

**Fig. 4:**
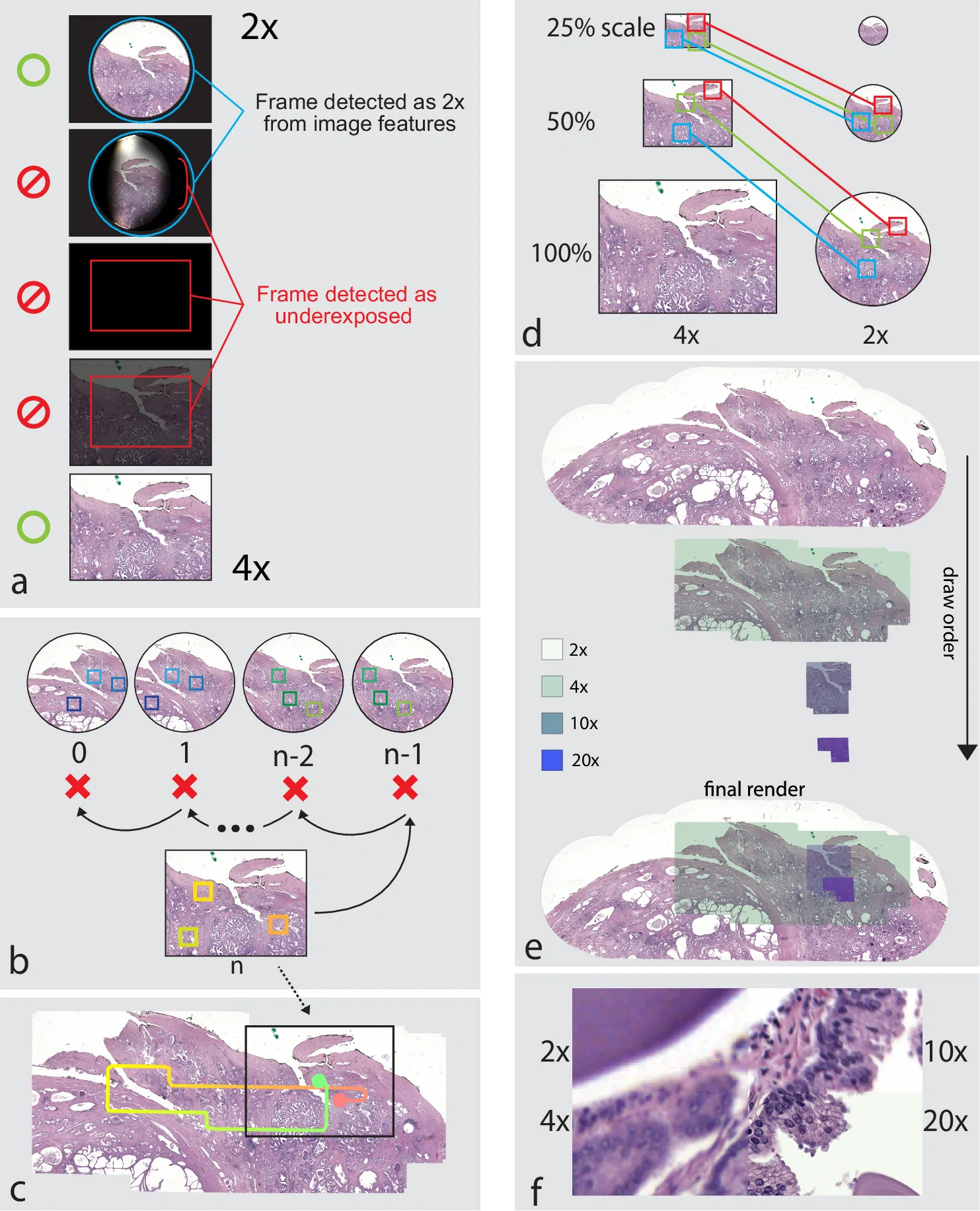
Multi-resolution slide images from unconstrained slide review. **a** When the pathologist changes objective lenses, underexposed (red) frames are detected and filtered out. **b** The first frame from the new magnification will attempt to match to each previous frame of the old magnification using single resolution ORB features (orange and yellow). **c** After the frame fails to match to any previous image, it becomes the root of a new connected component and each subsequent frame to pairwise match to a member frame of this component also becomes a member. Path shows the center location of frames as the component was built from red to green with the newest member frame outlined in black. **d** Once a frame becomes a member of a component, SIFT features are extracted (red, green, blue) and multiresolution matching is attempted against all other frames. When a root image succeeds at multiresolution matching, an initial estimate of scale offset and offset for its component is set. **e** Connected components are rendered with a priority ordering of higher resolution components (blue) over lower resolution components (grey) to create Multi-Resolution Slide Images (MRSI). **f** Shows the dramatic difference in clarity that can be made available by microscope review of a slide region using different objective lens magnifications.

We determined that changes in objective lens magnification could be detected and quantified using the real-time registration process. Practically, when a pathologist switches microscope magnification levels while the HistoCAM process is running, incoming frames will attempt to match against all previous frames, as seen in Fig. 4. Since ORB features are extracted at a single scale, frames collected at a new objective magnification cannot be matched to previous frames because of scale differences. We devised a method such that the first frame to pass quality assurance after the objective lens change is designated a root image and begins a new connected component in the mosaicking process. SIFT features are extracted from the root image and used for multiresolution matching against each member frame of every component. While using SIFT features is computationally expensive, the method’s ability to match across scale and the availability of efficient GPU implementations^29^ for extraction and matching of keypoints makes it a practical solution for frame matching across objective lens changes. For the initial scale and offset estimate of a component, the root image averages across all multiresolution pairwise matches it obtains, although a single match is sufficient. Incoming frames either spawn a new connected component or join an existing one, with the entire component rendered according to the scale and offset parameters determined by SIFT. This process can be repeated for any number of objective lens changes, in arbitrary sequence, including large magnification jumps (e.g., 2X to 40X). New connected components for each magnification level are stored as a tiled image pyramid and rendered in order of scale, such that the highest resolution data available in any area is displayed in the final Multi-Resolution Slide Image. Supplementary Movie 2 demonstrates real-time, multi-resolution image compositing and rendering using this approach, and Supplementary Fig. 3 shows example MRSI’s with overlaid magnification maps from expert pathologist review of radical prostatectomy slides. The reconstructed MRSI data and the associated spatiotemporal telemetry are stored using a modular architecture. The composited image pyramid serves as the primary (stable) image layer, while telemetry data, including frame timestamps, magnification level, spatial registration coordinates, and navigation trajectories, are stored as linked metadata layers referenced through shared spatial and temporal indices. This structure allows the image content, telemetry data, and derived metadata to be processed and versioned independently while preserving an immutable visual record of the captured microscopy session. Such modularization facilitates interoperability with downstream computational pathology pipelines and enables flexible integration of additional metadata layers – such as user annotations, segmentation masks, AI outputs, or provenance information – without modification of the underlying image data.

### HistoCAM data tell the story of glass slide diagnosis

**Fig. 5:**
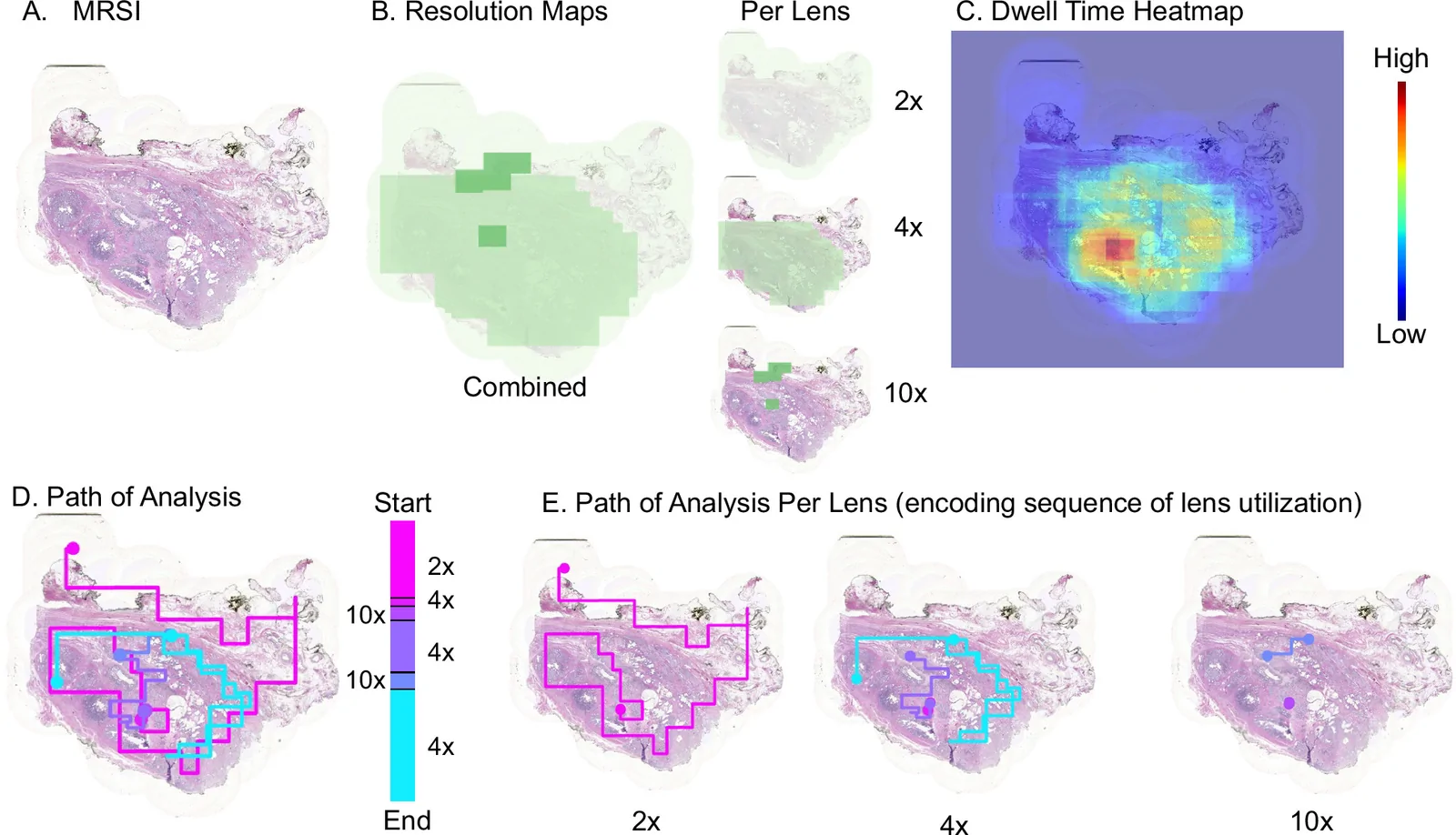
HistoCAM offers comprehensive spatiotemporal telemetry data. **A** Multi-Resolution Slide Images (MRSIs) output by the HistoCAM system. **B** Resolution maps can be generated to show which magnifications contributed to the diagnosis (light green represents low magnification objectives, dark green represents higher magnification objectives). Spatiotemporal data derived from the registration and compositing process enables generation of **C** dwell time heatmaps (high attention areas in red with a gradient to low attention areas in blue) and **D** time-and-magnification-encoded path of analysis for all lenses combined (magenta at the beginning of the recorded path and cyan at the end) or **E** per lens (magenta for 2X, cyan and blue for 4X, blue and purple for 10X), to show further insight into the process of diagnosis.

An important feature of our approach is that, in addition to producing multi-resolution digital slide images, the video-mosaicking registration provides spatiotemporal relationships between frames, including the eyepiece field-of-view location, magnification, and dwell time. Because our platform does not require the pathologist to divert their attention from the eyepieces during glass slide review, these paths reflect natural search patterns across magnifications. These previously unavailable data on slide review enable visualization of the spatiotemporal search patterns that contribute to the pathologist’s diagnostic process (Fig. 5). In addition to static MRSI (Fig. 5A) and the associated magnification maps (Fig. 5B), which show the areas of the slide viewed at each magnification, spatiotemporal data are also collected that provide new insights into the review process. For instance, timestamps and registration coordinates for each frame allow us to see not only where the pathologist looked and at what magnification, but also for how long. The latter data can be represented in a dwell time heatmap over the specimen as in Fig. 5C. The overall path and sequence of the analysis can also be reconstructed, where the path of analysis is overlaid onto the HistoCAM MRSI and is color-coded according to the temporal sequence and objective lens used (shown in the legend of Fig. 5D).

Additionally, the search path data can be isolated for each objective lens used, enabling one to observe the search strategy of the user as a function of magnification level. These rich spatiotemporal data, combined with the image data from the MRSI and each captured frame, reveal which regions were most salient and the precise sequence of events leading to the diagnosis. Such data, acquired from the gold standard of pathology diagnostic workflows and previously unavailable, could be invaluable for (a) the study of factors contributing to the accuracy and variability of pathologic diagnoses and (b) the generation of copious amounts of image and user-interaction data useful for the development of a multitude of AI/ML-enabled computational pathology (CPath^30^) approaches. They can also be rendered in real-time for immediate review by pathologist users to inform quality assurance (for instance, ensuring that adequate attention was paid to areas of diagnostic importance). Supplementary Fig. 4 shows an example pathologist review of a radical prostatectomy section with HistoCAM in which review of the MRSI after diagnosis revealed an area that was not covered. Although in this case the diagnosis was unchanged after the pathologist returned to the slide to review this area, it demonstrates the potential for coverage or saliency checks in real-time during diagnosis which could reduce errors and intra-rater variability due to suboptimal review. In addition, when combined with a customized WSI viewer (HistomicsUI^31^) that tracks user interactions on digital slides, it is possible to compare search behaviors between glass (HistoCAM) and digital (WSI) workflows on the same slide (Supplementary Fig. 11, Supplementary Fig. 12, Supplementary Movie 3). In an example of how such a workflow could aid in diagnosis, Supplementary Fig. 5 shows an example where digital review of an initial HistoCAM slide session was used to inform a second HistoCAM review session to confirm details of a salient area.

### HistoCAM as a computer-enabled workflow uplift for the pathologist

HistoCAM is designed to integrate seamlessly into the pathologist’s workflow, providing digital assistance that supports routine tasks while enhancing accuracy and efficiency. Because the HistoCAM approach ambiently digitizes the gold-standard glass slide diagnosis, beyond the compositing and display of everything the pathologist has seen on the glass slide, HistoCAM also serves as a platform for digital interaction with the images that were created. As one example, when analyzing and diagnosing slides from patients who have undergone radical prostatectomies, typically between 50 and 150 slides need to be reviewed for a single patient, and a number of factors and measurements must be documented for the pathology report. For radical prostatectomies, one of the most important items noted in the pathology report is the Gleason ISUP Grade Group, which is determined by the sum and relative proportion of the Gleason scores for the two most prominent tumor architectural patterns. The pathologist must also estimate the total tumor volume and identify aspects such as the presence, location, and length of areas of positive tumor margins; the presence of certain morphological diagnostic and prognostic features such as extraprostatic extension, seminal vesicle invasion, perineural invasion, lymphovascular invasion, and the presence of certain morphological subtypes such as cribriform morphology; as well as make note of which slides contain tumor. To mark the presence and location of tumor, or other features such as locations of positive tumor margins, pathologists will often mark the slide manually with an ink marker. Once all of the slides are reviewed, the marked areas on the slide are then measured with rulers, calipers, or simple eyeballing to provide a rough estimate of tumor volume in the gland as a percentage of the total tissue area, the location and length of positive tumor margins, as well as to document which slides contain other diagnostic/prognostic features. This process is semi-quantitative at best, and completely manual, contributing greatly to interobserver variability^32^ and time required for the diagnostic review.

As a hybrid analog-digital approach, HistoCAM can make this process simpler, faster, and more accurate, as shown in Supplementary Fig. 6. Using the HistoCAM App software application, the pathologist has access to several tools for annotating the MRSIs generated in real-time during the glass slide review. Immediately after each slide is reviewed, using the point-and-click polygon annotation tool in the HistoCAM App, the pathologist can create polygons of any shape to annotate the location of the tumor, its diagnostic category, and its tumor area, the latter of which can be exactly measured since the size of each digital pixel in the composite image is known. Using the same method, the pathologist can outline the entire tissue area to calculate the exact percentage containing tumor on each slide. These values can then be compiled across slides to provide an accurate determination of tumor volume. The measuring tools in the HistoCAM App can also be used to assess features such as tumor distance from the margin and the total length of positive margins. These measurements are clinically relevant, as both values must be reported in the pathology record. Finally, user-created annotations and labels are stored digitally in real time as the pathologist reviews each slide. These records can then be synthesized at the end of the session to document all relevant findings from the case. By enabling digital measurement, documentation, and verification of all areas of interest during routine glass slide review, the system allows pathologists to work more efficiently while maintaining diagnostic accuracy.

### HistoCAM supports real-time AI tool integration

**Fig. 6:**
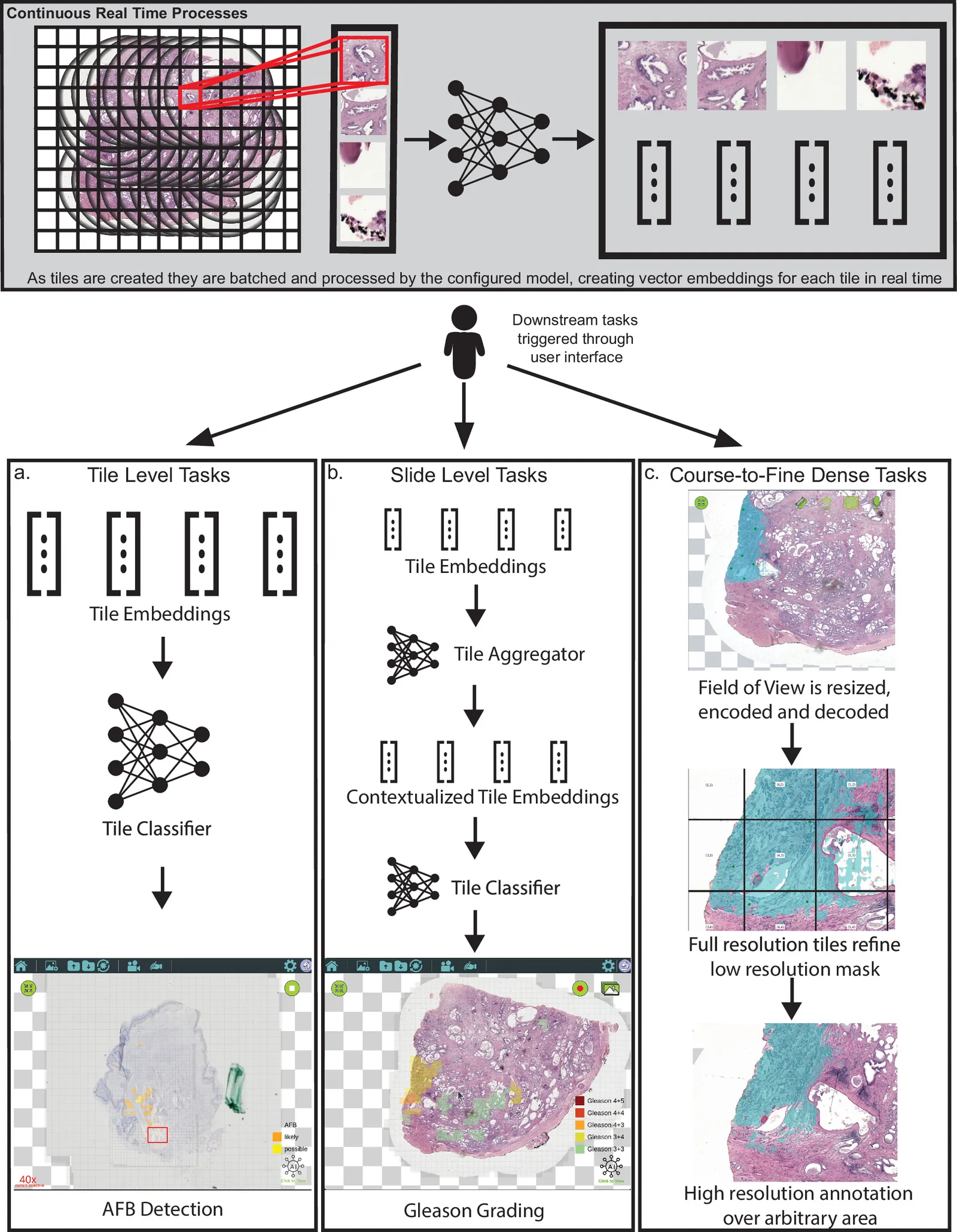
Continuous tile embedding and configurable downstream tasks in real-time using HistoCAM. As tiles are generated, they are immediately batched and embedded with the configured encoder. Updated tiles are re-embedded, replacing prior embeddings. **a** Tile-level tasks operate directly on individual embeddings without slide context. For example, PathDINO^34^ with a custom Multilayer Perceptron (MLP) classifier can identify acid-fast bacteria (AFB) within a single tile (AFB likely denoted in orange, AFB possible denoted in yellow). **b** Slide-level tasks aggregate embeddings across tiles. In the example shown, tiles are continuously embedded with the Prov-Gigapath^35^ encoder. When triggered by the user in the HistoCAM App, embeddings are passed to the Prov-Gigapath aggregator (a self-attention module that incorporates neighborhood context) before classification with a lightweight MLP (e.g., Gleason grading denoted in a gradient from Gleason 4 + 5> in dark red to Gleason Grade 3 + 3 in light green). **c** Coarse-to-fine dense tasks begin with a resized field of view treated as a single input tile. The output of the dense model (e.g., Segment Anything 2^33^ by Meta) is partitioned to match the corresponding full-resolution tiles. This low-resolution contextual output is then refined by a dense model at tile resolution (shown in blue). Supplementary material includes real-time demonstration videos for each task.

HistoCAM also provides an opportunity to integrate AI enhancements at both the slide level (context-aware) and the FOV level into routine glass-slide review, including semantic segmentation and classification tools powered by recent pathology foundation models (Fig. 6). In contrast to prior integrations of AI into the glass slide workflow^11^, HistoCAM enables analyses at the whole slide level, and potentially learning from and leveraging the spatiotemporal data gleaned from the diagnostic process. To provide this capability, we developed the HistoCAM software architecture such that as images are composited into an image pyramid, in parallel to the current process of real-time MRSI creation, each tile can also be embedded by a configured tile encoder model. Tile embedding is processed in batches to create feature vector representations of each tile, which can later be classified, aggregated, or used for dense tasks like segmentation. Tile encoders used in Fig. 6 include the SAM2^33^ ViT Large Image Encoder/Decoder, PathDINO^34^ foundation model, and Prov-Gigapath^35^ foundation model. Fig. 6 details two example workflows for real-time diagnostic inferencing, the first being Acid Fast Bacteria (AFB) identification and the second being assisted diagnostic inferencing for the purpose of Gleason / ISUP grading on radical prostatectomy slides. The first example uses PathDINO with a custom Multilayer Perceptron (MLP) classifier to detect Acid-Fast Bacilli (AFB) in a sputum swab slide. This example, shown in Supplementary Movie 4 utilizes HistoCAM’s tile-level inferencing pipeline as shown in Fig. 6a. A user broadly sweeps the slide at 4X resolution and triggers tile classification through the user interface. Tiles are then highlighted as AFB-possible, AFB-likely, or not at all. The user then changed objectives to 10X and broadly swept an area with a significant density of highlighted 4X tiles. Automatic, real-time embedding of tiles happens again as the 10X composite is made, after which the user again triggers tile classification, producing highlighted tiles at 10X with the same class labels. The user then centers the FOV on a 10X tile highlighted as Likely and switches objectives to 40X. With this FOV, the user is able to positively confirm the presence of AFB in the sample. Detecting the presence of AFB represents an example of a tile-level task where a determination can be made for each tile independently of any other tile. This example also highlights the way HistoCAM can be used in tandem with conventional microscopy to expediate tedious searches.

The second example is Gleason grading of a radical prostatectomy slide, which utilizes HistoCAM’s slide-level inferencing pipeline (Supplementary Movie 5). This example uses the Prov-Gigapath foundation^35^ model with exposed token embeddings in the last layer of the slide aggregator. The user views the slide as normal through a low-power objective typical for Gleason grading (in this example, 2X). As the composite is created, tiles are embedded using Prov-Gigapath’s DINOv2^36^ based tile encoder. When the user is ready, they trigger classification in HistoCAM UI, which takes all existing tile embeddings as input to the slide aggregator. The contextualized tile embeddings are then classified with a custom MLP to produce a final Gleason score for each tile. This example in particular highlights the value of HistoCAM in tasks like ISUP grading, which require total area and proportion calculation of Gleason grades over a whole slide, a task that is difficult to do when limited to a microscope’s FOV. It is important to note that in the examples of AFB identification and Gleason/ISUP assisted diagnostic inferencing, models have been fine-tuned on a very limited data set, and we would not expect them to generalize well to diverse data sets. However, these proof-of-principle examples are intended to demonstrate the real-time capabilities of the HistoCAM system architecture with a variety of configured models, and we believe that the creation of generalizable models for these tasks and others is possible utilizing data recorded by HistoCAM.

HistoCAM also offers a unique value proposition as an engine for the rapid generation of tile-level annotated training data, shown in Supplementary Movie 6. To accomplish this, we integrated Meta’s Segment Anything Model 2 (SAM2) into the HistoCAM application as a method for rapid annotation and labelling of ROIs (this feature also supports the workflow uplift described in the prior section). The user can navigate to the annotation feature of the HistoCAM App and select the segment icon, then, using mouse clicks as embedding prompts, quickly create high-quality labeled annotations of tissue features of note. These segmentations of tissue features can then serve two purposes: the first is free tile-level annotations derived from slide-level segmentations, and the second is an aggregation of notable features for record-keeping when noting important disease subtyping. HistoCAM achieves free tile-level annotations by contrasting the masks created by SAM2 with the underlying tile grid of the composite. By looking at the 256×256 tiles which make up the SAM2 tiles, whose image coordinates are already known, we are able to rapidly create bags of pre-embedded tiles with the associated label created during the segmentation process. This cuts out the tedious and time-consuming processing required for applying tile-level annotations directly by hand (which lack context of other tissue in the area) or the need to break down slide-level annotations. HistoCAM can thus serve as a data factory for both generalized and task-specific foundation models that are no longer limited by the need for high-resolution image scans, creating bags of annotated and pre-embedded digital training data nearly as fast as the slides can be reviewed by the pathologist using the microscope. The ambient datafication of behavior-informed datasets created by HistoCAM-documented diagnostic reviews presents a potential source for novel corpora for training new multi-scale foundation models, pathologist-like analysis strategies, or rapid fine-tuning for task-specific models.

### Deployment on a compact edge AI computing system

As previously described, one of the major barriers for the adoption of digital pathology is the substantial financial investment required to purchase, maintain, and operate high-throughput FDA-approved whole slide scanners. HistoCAM is intended to provide access to key benefits of digital pathology without requiring a whole-slide scanning system; however, we recognize that the cost and footprint of HistoCAM hardware must also be minimized to promote broader adoption. To reduce costs as well as physical footprint, we deployed the HistoCAM system on a compact, relatively low-cost unified memory computer (NVIDIA DGX Spark). We verified that this system is capable of operating the HistoCAM hardware and supporting real-time image acquisition, compositing, and AI inference at substantially lower cost and physical footprint compared to our original custom workstation (Supplementary Movie 7).

Specifically, the DGX Spark architecture integrates CPU and GPU resources within a shared memory space (128 GB unified system memory), which reduces data transfer overhead between the CPU and GPU processing units and mitigates bottlenecks commonly encountered in CPU-GPU systems with discrete memory. By eliminating the need to copy image data between separate memory pools, the system enables efficient handling of large image datasets and supports GPU-accelerated computation across the full memory capacity. In addition, the platform provides a native CUDA-enabled environment, facilitating integration of AI models and image analysis pipelines within the HistoCAM framework. Together, these characteristics support a compact and scalable computational implementation that may lower barriers to deployment in resource-constrained settings.

## Discussion

In this paper, we present HistoCAM, a hybrid analog-digital pathology platform based on a simple insight that the video feeds from cameras installed on clinical microscopes can be used to build complete digital records of clinical slide reviews in real-time, when appropriately configured to comprehensively sample the entire microscope field of view with adequate spatiotemporal resolution and data-efficient compositing. As the pathologist moves the stage at normal speed while looking exclusively through the eyepieces, the system streams high-resolution full-FOV video fast enough to eliminate motion blur, automatically detects every objective-lens change, and stitches the resulting frames into a seamless multi-resolution slide that spans 2X to 40X. While it composes the mosaic, HistoCAM concurrently records how long each region and magnification is viewed, and in what sequence, thereby creating a spatiotemporal log of the diagnostic search and completing a pathologist-computer feedback loop during traditional glass slide review. Key to our approach were human-centered design and robust engineering efforts to make the process as efficient and unobtrusive to the gold-standard workflow as possible, to promote likelihood of eventual adoption. This was achieved via the use of a trinocular beamsplitter head and a large-format, high-space-time-bandwidth camera sensor, which faithfully captured the full area and optical detail that is observed by the pathologist through the eyepieces during the slide viewing session. Pathologists are able to move the sample at any speed and in any pattern without fear of gaps in the image and can leave the microscope to attend to other tasks during a recording session without fear of data pileup. This ability means that the pathologist does not need to look away from the slide to monitor the image acquisition on a computer screen to ensure full coverage; and in fact, as we showed, real-time HistoCAM previews can provide the opposite assessment by alerting pathologists to unobserved (or minimally-observed) areas, by providing images in real-time and overlaying the magnification usage and dwell time maps. This high information-bandwidth system presented a particular engineering challenge due to the large amount of image data that must be efficiently handled in real time. Parallel GPU-accelerated processing, efficient and scale-aware graph-based compositing, intelligent sampling and memory usage, and low-overhead programming provided the necessary system performance.

Due to our unobtrusive approach, the data produced by HistoCAM are reflective of the information leveraged by the pathologist to render a diagnosis on the glass slide. Recent work in the field of computational pathology has addressed the need to take more efficient, pathologist-like approaches for post hoc AI analyses after WSI collection^37^, including frameworks which involve pathologist-AI collaboration on the digitized slides^38^. Rather than such post-hoc approaches, HistoCAM builds this efficiency in from the data generation step, while also natively including the collaboration and interaction with expert pathologists during the standard diagnostic glass slide review itself. The resulting MRSI’s represent all regions of the specimen that were actively examined at diagnostic resolution, but prevent excessive high-resolution sampling of non-salient areas that typically contribute to data bloat in uniformly scanned WSIs. In our dataset, this targeted acquisition led to raw MRSI file sizes that are 5-10% (on average) of the file sizes for WSI slides scanned at uniform 20X magnification (~0.5 μm per pixel), the latter of which are approximately four times smaller than the 40X (~0.25 μm per pixel) resolution recommended for primary diagnosis. This indicates that HistoCAM composites (created during the gold-standard diagnostic procedure) may be 40 to 80 times smaller than equivalent WSIs needed for digital diagnosis. While this selective sampling results in a sparser dataset than full-resolution WSIs, it offers a more efficient representation of diagnostically relevant information while still ensuring complete slide coverage. Indeed, the assumption that more data necessarily equates to more diagnostic value does not always hold; pathology has long been shaped by inter-rater variability and subjective prioritization of visual cues. In fact, HistoCAM’s real-time feedback and quality assurance capabilities introduce the potential for reducing diagnostic variability and enhancing accuracy during conventional glass slide review by enabling users to check their work or to easily collaborate for second opinions. Moreover, by significantly reducing the cost and complexity of digitization, HistoCAM may enable large-scale capture of cases that would otherwise remain undigitized under current clinical WSI adoption rates. In doing so, it may yield an overall increase, not decrease, in the volume and diversity of computational pathology datasets available for machine learning and clinical implementation, including unique telemetry data gained for free during the diagnosis/digitization process. To our knowledge, there are no other technologies that record or visualize magnification levels, paths of analysis, and dwell time of pathologists reviewing glass slides at the microscope using unaltered diagnostic routines.

User-tracking telemetry has been extensively studied in radiology to understand how radiologists search images, interact with viewing systems, and make diagnostic decisions. The most widely used approach is eye-tracking, which records fixation locations, scan paths, and dwell times to characterize visual search behavior during image interpretation. These studies have shown that diagnostic errors often arise from search or recognition failures and that expert radiologists exhibit more efficient gaze patterns than trainees^39–41^. Eye-tracking data has also been incorporated into machine-learning pipelines to guide model attention toward clinically relevant image regions^42^. More recently, telemetry frameworks combining gaze data, interaction logs, and workflow context have been proposed to measure how AI systems influence radiologist behavior and decision-making^43^.

These findings suggest several potential roles for telemetry in digital pathology. Navigation traces could be used to learn diagnostic search trajectories, enabling models to predict which regions of a slide should be examined after the initial low-magnification overview^44^. Telemetry may also enable workflow-aware evaluation of AI systems, quantifying how AI assistance alters search patterns, efficiency, and time-to-diagnosis^43^. Finally, interaction data may provide a form of weak supervision for AI training, generating attention maps or evidence paths based on signals such as magnification changes, navigation paths, and dwell times. Such signals can be derived from digital slide viewers through viewport tracking or eye-tracking. However, as shown in Supplementary Figs. 4 and 5 and Supplementary Movie 3, viewing behavior differs substantially between glass-slide microscopy (obtained via HistoCAM telemetry) and digital slide review (obtained via viewport tracking), even for the same pathologist reviewing the same slide on the microscope vs. digitally. Because digital WSI slide viewers rely on general-purpose input devices (i.e., keyboard, mouse), interaction logs may include noisy signals from incidental user actions, whereas HistoCAM microscope navigation requires more deliberate movements (e.g., objective lens changes, physical slide movements) that may more directly reflect diagnostic intent. Nevertheless, both modalities provide rich telemetry data that may enable modeling of pathologists’ stepwise diagnostic reasoning and support the development of human-aligned computational pathology systems, an area of growing recent interest^37,45–47^.

In addition, we provided examples of HistoCAM MRSI’s suitability for digital workflow-uplift tools, and AI-at-the-microscope applications for pathologists who may not have access to an integrated digital pathology lab. In doing so, adoption of HistoCAM could demonstrate significant industry value and patient impact by expanding the potential market for AI-enabled decision support tools. Notably, application of models trained on specific patient populations may have lower performance when applied to new patient populations, or edge cases that they were not trained on. While HistoCAM is uniquely suited to support context-aware (i.e., slide-level) anatomic diagnostic pathology workflows in real-time, it still retains the ability to support context-agnostic pipelines as well (such as in intensive search tasks in cytology, hematopathology, and diagnosis of infections such as malaria or tuberculosis) in which images are considered solely at the patch or ROI-level. Note that HistoCAM is, in principle, compatible with any type of microscopy, including polarization and the use of high-NA oil immersion objectives used for cytology. In addition, by rapidly generating multi-resolution slide images with pre-embedded and annotated tiles during standard clinical diagnosis without slowing down the procedure, HistoCAM can change how machine learning datasets are curated at scale. Such pipelines and data could be useful for generating corpora of image, interaction, and annotation data for free during glass slide clinical diagnosis, which could be useful for training next-generation foundation models especially suited for pathologist-like image analysis, or for rapidly fine-tuning existing foundation models^37^ for specific tasks valuable to the organization. The ability to support both real-time computational assistance and fast digital collaboration to improve diagnostic accuracy in a larger dataset will be studied in future work. This work demonstrates the potential of the HistoCAM system to connect digital pathology capabilities to the current analog clinical pathology workflow, and to provide comprehensive human-machine interaction datasets that could be valuable resources for future research and clinical decision support.

Accordingly, HistoCAM extends beyond digitization to datafication, by virtue of its comprehensive user telemetry layer. Datafication has created step-change advances in areas such as personalized media recommendations, shipping logistics, energy efficiency, public health surveillance, personalized health monitoring, and other domains. In each case, the advance was not due solely to digitization, but also to the systematic capture of process-level data: analyzable signals that can power new tools, optimizations, and applications. HistoCAM extends digitization into real-time datafication of the diagnostic act itself, on the workflow used in the vast majority of diagnoses performed today. The resulting telemetry provides a new layer that complements pixel-level image data: these process-level signals can inform models that learn not only what diagnosis was rendered, but also how experts arrived there. Additionally, such data may enable quality assurance tools that highlight under-sampled regions, support adaptive training platforms that mirror expert visual search strategies, and unlock richer AI-pathologist interactions at the microscope. As in other domains, the shift from digitization to datafication in pathology may represent an inflection point that transforms computational pathology into a prospective, workflow-integrated, and process-aware discipline compatible with the most prevalent workflows of the day.

## Methods

### System design

To capture the pathologist’s microscope analyses, we attached a large-format CMOS camera (Sony IMX342/FLIR Oryx Color 31MP 10 GigE) on two separate microscope builds. The first was a Nikon Eclipse Si upright microscope equipped with 2X (Nikon CFI Plan Apochromat Lambda 2X Objective Lens, NA 0.1, WD 8.5 mm, FN 25 mm), 4X (Nikon CFI Plan Fluor 4X Objective Lens, NA 0.13, WD 17.2 mm, FN 25 mm), 10X (Nikon CFI Plan Apochromat Lambda 10X Objective Lens, NA 0.45, WD 4 mm, FN 25 mm), 20x (Nikon CFI Plan Apochromat Lambda 20X Objective Lens, NA 0.75, WD 1 mm, FN 25 mm), and 40X (Nikon CFI60 E Plan Achromat 40X Objective Lens, NA 0.65, WD 0.65 mm, FN 20 mm) objectives. The microscope’s light source was split to provide 20% to the eyepieces (Nikon CFI 10x Eyepieces, FN 22 mm) and 80% to the camera’s sensor simultaneously using a Nikon C-TT trinocular tube, with no additional adaptor magnification between the camera and the microscope optics. This build had the limitation that the substage condenser was fixed, causing vignetting for the 2X objective lens only. The second microscope build was a Nikon Eclipse CI upright with 2X (Nikon CFI Plan Apochromat Lambda 2X Objective Lens, NA 0.1, WD 8.5 mm, FN 25 mm), 4X (Nikon CFI Plan Apochromat Lambda 4X Objective Lens, NA 0.2, WD 20 mm, FN 25 mm), 10X (Nikon CFI Plan Apochromat Lambda 2X Objective Lens, NA 0.45, WD 0.17 mm, FN 25 mm), 20X (Nikon CFI Plan Apochromat Lambda 2X Objective Lens, NA 0.8, WD 0.8 mm, FN 25 mm), and 40X (Nikon CFI Plan Apochromat Lambda 2X Objective Lens, NA 0.95, WD 0.17–0.25 mm, FN 25 mm) objective lenses. This build was equipped with a swing-out substage condenser, which, when removed for the low magnification lenses, eliminated the vignetting problem and enabled full coverage of the eyepiece FOV at all magnifications. Use of the substage condenser to improve resolution for higher magnification lenses did not result in vignetting.

Initial research was performed using a dedicated workstation consisting of an ASUS Pro WS WRX80E motherboard with multiple PCIe and M.2 slots to support high-bandwidth cameras and other hardware, running an AMD Ryzen Threadripper PRO 5986WX (24 core). We chose 256 GB of quad-channel DDR4-3200MHz RAM to support the buffer and furthermore chose a 4TB M.2 NVMe SSD drive as a primary acquisition drive. An additional 4TB SATA SSD is mounted for permanent data storage. For the purposes of compositing and the creation of vector embeddings in real-time, an NVIDIA GeForce 3090 TI and NVIDIA GeForce RTX 4090 were used; compositing was performed on the 3090 while the 4090 performed tile embedding and inferencing tasks. The platform (hardware and software) was subsequently fully deployed on a compact, shared CPU-GPU memory computer (NVIDIA DGX Spark), replacing the earlier workstation.

### Camera spatiotemporal sampling

We chose a camera that both properly sampled the optical resolution of the microscope at all magnifications, satisfying the Nyquist-Shannon criterion^48^, and that also provided sufficient temporal sampling to accommodate unconstrained slide movement during analysis. With our current camera sensor (Sony IMX342 Color, 6464 ×4852 pixels), the pixel size is 3.45 microns. Supplementary Table 1 contains a list of Nikon objective lenses tested in the HistoCAM prototypes, their corresponding diffraction-limited resolution, optical FOV, and SBP characteristics using CFI 10X eyepieces with 22 mm FOV, and the corresponding HistoCAM digitization system characteristics including system resolution, eyepiece FOV coverage, and SBP coverage.

### Camera frame rate analysis

We determined the required camera frame rate according to the maximum stage expected translation speed in combination with the frame size. The camera had to quickly acquire images such that there were no gaps in the final stitched mosaic due to excessive inter-frame movement of the slide. The frame rate had to be high enough to ensure a minimum level of overlap between adjacent frames at maximum stage speed, which we conservatively set as 20% for sparse feature matching. We measured maximum expected stage speed (7 mm/s) by recording a pathologist reviewing radical prostatectomy specimens with a high frame rate (200 FPS) camera (FLIR Blackfly S, CMOS, 0.4 MP) (Supplementary Fig. 8). We found the displacement between successive frames in the video stream using SIFT/RANSAC^49^, described in detail below, then calculated stage translation speed based on the frame’s millisecond-resolution timestamp. The minimum required frame rate is then a function of stage speed. The maximum stage translation displacement (measured in millimeters) which maintains at least 20% overlap is given by the equation:

1 $${{\rm{Displacement}}}={{\rm{frame}}}\; {{\rm{width}}}\; {{\rm{x}}}\, (1-0.2).$$

Therefore, the minimum frame rate to ensure the specified overlap is:

2 $${{\rm{Frame\; Rate}}}\,\left({{\rm{FPS}}}\right)={{\rm{stage}}}\; {{\rm{speed}}}\,{x}\frac{2\,{{\rm{frames}}}}{{{\rm{displacement}}}}$$

Using these equations, for our chosen camera sensor (Sony IMX342, APS-C format), we found that the minimum frame rate to ensure a gap-free video stream using all objective lenses was 7 FPS, or a space-bandwidth-time product (SBTP) of 154.6 megapixels/s, well below the maximum full-frame speed of the FLIR Oryx 31MP camera of 26 FPS (SBTP of 815 megapixels/s). Video frames were captured in Bayer RG8 Raw and debayered in real-time. The resolution of each frame was 6464 ×4852 pixels.

### HistoCAM desktop application

A user-friendly desktop application (HistoCAM App) was built using C + + to house HistoCAM’s image compositing technology, using JUCE^50^ and OpenCV libraries, as well as to handle recording via camera control. The front-end application’s primary function is to query the image pyramid that HistoCAM creates for the appropriate tiles to display given the user’s digital FOV (see the following section for a description of the image pyramid structure and its creation). HistoCAM App contains built-in functions for digital annotation, labeling, area and length calculation. It also serves as the user-interface for AI-at-the-microscope tasks, such as AI-assisted segmentation or classification tasks, using a pre-configured user-selectable model. These features are shown in Supplementary Data Fig. 2.

### Multi-resolution recording and compositing in real-time

Users initiated a recording session using the HistoCAM App. Image frames were acquired continuously and transferred from the camera buffer into system memory. White balancing was performed on the camera utilizing a fixed 1.5-to-1 ratio of red channel gain to blue channel gain. Upon transfer, an image object was instantiated to encapsulate frame-specific metadata and pixel data. A loader job was then created to perform initial processing and assigned a priority equal to the frame index. All jobs were submitted to a multithreaded queue that remained active throughout the runtime. Jobs were scheduled by increasing priority value, such that lower indices were processed with higher urgency.

Loader jobs performed debayering using a standard RGGB interpolation pattern through OpenCV^51^, followed by a quality control analysis to assess blur and exposure. Frames failing the blur and exposure criteria were discarded, as well as three frames on either side (i.e., before and after). This ensured that additional blurry and underexposed or overexposed frames that often surround objective lens changes were discarded as well. For frames that passed quality control, cropped and down-sampled registration images were generated to reduce computational load. From each registration image, single-scale ORB features were extracted on the CPU. Processed full-resolution frames were kept in memory until they were deemed unfit for compositing or written to disk, then transferred to the GPU to maintain low memory usage. Upon completion, each loader job enqueued a matcher job with priority incremented by 20 to allow temporal separation from initial processing and to ensure any given frame likely to match has had sufficient time for its features to be extracted before attempting a match. Variability in thread scheduling occasionally caused loader jobs for higher-index frames to complete before those of preceding frames. To accommodate this, a configurable delay was introduced before initiating the matching of subsequent frames.

Matcher jobs performed pairwise feature matching between frame *n* and preceding frames (*n*−1*, n*−2,…,0*)*. Matching was implemented using single-scale ORB descriptors and brute-force Hamming distance computation between frames that met matching requirements. Frames that failed to match any prior frame were then designated as roots of new components. For each successful match, a registration job was created with priority incremented by 20, as a method for deferred scheduling. This ensured the completion of contiguous pairwise matches prior to registration. Registration jobs then determined component membership and absolute coordinates by chaining pairwise matches together. If the predecessor’s coordinates and component membership were already established, these were propagated to the current frame, registered as a dependent via a callers-waiting (index of previously unmatched frames) mechanism, and exited the queue. Once the predecessor completed registration, it propagated the necessary metadata to all dependents.

Cross-component matching followed a similar approach. We assume that the user will begin with the 2X objective lens (this is configurable to start at any magnification level). The first quality frame of each component was accepted. After the first frame was accepted, the next frame with sufficient pixel distance from the last accepted frame was accepted, saved to disk, and its raw buffer was copied to the GPU. Frames that were not accepted were discarded and never saved to disk. Once a frame was on the GPU, it was debayered again from RAW and flat-field corrected. For flat field correction, frames were divided by a calibration image (a focused image of the light source with no slide mounted) taken at each respective magnification level. It then underwent SIFT feature extraction. If the frame was the root of a new component, its SIFT features were matched against the SIFT features of every other frame. Based on the SIFT registration of the root image, the entire component was given a scale and offset from the first component, which was anchored at the origin in pixel space. Frames designated as new component roots generated cross-component matching jobs with priority zero for immediate processing. These jobs first attempted multiscale SIFT matching against frame *n*−1. Once matching succeeded, the job requested the coordinates and component metadata of the predecessor. If either the coordinates or component metadata were unavailable, a cross-component dependency was recorded, and the job exited the queue. Once registration was completed, the predecessor disseminated the required information to trigger downstream operations, including component scaling and compositing. Throughout this process, a dedicated composite manager thread continuously monitored the queue of frames awaiting graph-based compositing. Frames were sorted by component and passed to the component update routine. Typically, a single frame was processed per cycle, although batches were used during component reconstruction. Candidate frames for compositing were inserted into the Voronoi diagram, and the corresponding Voronoi face area was calculated. Frames whose Voronoi face area was less than 20% of the frame area were discarded as well. Accepted frames retained their Voronoi face polygon as a copy-mask for compositing, as shown in Fig. 3.

Composite images were stored as non-contiguous tiles to avoid reallocation during incremental updates and to facilitate efficient downstream AI applications. After compositing, each updated tile was recursively downsampled by a factor of two along each dimension until a minimum resolution was reached. This generated an image pyramid that supported scalable rendering. During rendering, the viewer dynamically queried tiles within the current viewport and retrieved the lowest-resolution tiles compatible with the current zoom level. As the user zoomed in, higher-resolution tiles were subsequently loaded and displayed. Tiles belonging to higher-magnification components were prioritized over lower-resolution components, occluding them as appropriate during rendering. Final composite images can be exported as pyramidal Zarr files for downstream analysis or reloading in separate viewer applications (such as HistomicsUI).

### Image alignment

HistoCAM incorporates a system for realigning the composite image, which can be configured on or off by the user. Because the absolute coordinates of contributing frames are determined by pairwise chaining, visibly detectable stitch lines can exist in the composite image, particularly at loop closures. We quantify this error as the deviation between the difference of the absolute coordinates of two frames and their directly calculated pairwise homography. In our experience, this error metric rarely exceeds 80 pixels and averages about 18 pixels across composite images, typically on the order of 10-100 megapixels.

To reduce this error, we incorporated a robust bundle adjustment solution with an efficient GPU implementation^52^. In bundle adjustment, keypoints are matched across all frames in which they appear. Keypoints themselves are denoted as landmarks and their positions in worldspace become degrees of freedom. Each frame observes some set of landmarks in its own respective frame space, the x-y coordinates of which are taken as constraints. A non-linear system of reprojection errors is created which tracks the difference between where landmarks were observed in frames and where they should have been observed in frames given the positions of each frame and landmark in worldspace. Because this system is intended for general use in scene reconstruction, it enables additional degrees of freedom than our problem requires, including rotation of camera frame perspectives around x, y, and z. These parameters introduce strong non-linearity into the system and, in our experience, lead to numerical instability when only one system is used to place all frames, regardless of magnification. To avoid this issue, we set up and solve a bundle adjustment problem for each magnification, creating well-aligned components. We then treat each component as a single frame and set up and solve a final system to arrive at corrected scaling and alignment between components. We have found that this technique reduced maximum alignment error to nine pixels across our test set.

The time cost of the implementation is also relevant for practical use of our system, and we find that we can align and rebuild a composite image that consisted of four objectives (2X, 4X, 10X, and 20X) and 215 accepted frames in about eight seconds. The major bottleneck in this process is loading frames back to the GPU from disk so their data can be recopied to image pyramid tiles. By beginning this load process while still setting up the bundle adjustment system, we can effectively parallelize the problem.

### Diagnostic inferencing and AI applications in real-time

As tiles in the image pyramid are updated, they are immediately batched for embedding with the configured model. Our inferencing pipeline is highly configurable, but in general consists of tile embedding followed by tile-level classification, or tile aggregation for the purpose of slide-level classification. However, tile aggregation is optional and often not appropriate depending on the task. Tile-level tasks are those where tiles can be classified at the tile-level by their non-contextualized embeddings. In these tasks, tiles are embedded by a configured model. A user can trigger these embeddings through the application at any time to be classified by a configured classifier model, and their predicted labels are displayed as overlaid shading on each tile.

On the other hand, slide-level tasks expand upon tile-level tasks to create a pipeline where predictions are made across the entire slide from an aggregation of tile embeddings. Tile classification may still be the target, but it depends on both the tile’s position within the slide, as well as the tile’s feature embedding. In this pipeline, tiles are still embedded by a configured tile encoder model continuously as they are created for the image pyramid. At any time, the user can trigger slide-level inferencing using either a configured report model or a configured tile aggregation model. If the former is configured, a model (such as PRISM^53^) can take the existing tile embeddings as input and produce a text report of the slide. This is highly variable, and the text report can be as simple as a single slide-level score, as is the case with models that predict slide-level ISUP grades by classifying a CLS token. If the latter is configured, existing tile embeddings at the time of user-triggered slide-level inferencing are passed through a configured aggregator which must expose the tokens in its final layer. The tokens exposed after aggregation are understood to be contextualized versions of the input tile embeddings and are then classified with a configured classifier model. Their predicted labels are then displayed as in the tile-level task pipeline. See Fig. 6 and Supplementary Movies 4 and 5 for a realized demonstration of these pipelines.

### User-directed AI segmentations

To accelerate the annotation process, we employed clustering-driven segmentation methods as an alternative to manual polygon drawing. This approach substantially reduces the number of user interactions required, thereby providing a faster and more user-friendly annotation workflow. For segmentation, we used the Segment Anything Model 2 (SAM2) ViT-Large image encoder and decoder, deployed as TensorRT engines converted from ONNX models. Our current implementation is restricted to single-resolution composites. Because the native SAM input size (1024 × 1024 pixels) is considerably larger than that of most vision foundation models (typically 224 × 224 pixels), a single composite can generally be tessellated into fewer than 200 SAM tiles. This eliminates the need to embed tiles in real time. Instead, we realign and rebuild the composite image prior to embedding, thereby minimizing stitch errors that could otherwise degrade feature extraction.

Our annotation workflow follows a coarse-to-fine strategy. As users place foreground and background clicks, we record the minimum and maximum x–y coordinates of the viewed region. When segmentation is triggered, these coordinates define a bounding rectangle that is rescaled to 1024 × 1024 pixels. Along with the transformed click locations, this input generates an initial low-resolution segmentation mask spanning the selected region of the slide. The mask is then resized to full resolution and partitioned across the corresponding SAM2 tiles. Full-resolution tile embeddings, together with their portions of the coarse mask, are provided to the SAM2 decoder, which refines the coarse prediction into a high-resolution segmentation mask. This approach allows users to annotate naturally across arbitrarily large areas while still obtaining precise, full-resolution segmentations.

In practice, the entire process, including realignment with our bundle adjustment implementation, tessellation into 1024 × 1024 tiles, and embedding with the SAM2 encoder, requires less than seven seconds for a prostate resection slide composited at 2X. If embedding is still underway when a user begins annotating, tiles within the active coarse region are prioritized so that their embeddings are available for refinement as early as possible. This scheduling ensures that interactive segmentation remains responsive, even on very large composites. See Fig. 6 and Supplementary Movie 6 for further explanation and demonstration.

## Supplementary information

Supplementary Information

Description of Additional Supplementary Files

Supplementary Movie 1

Supplementary Movie 2

Supplementary Movie 3

Supplementary Movie 4

Supplementary Movie 5

Supplementary Movie 6

Supplementary Movie 7

Transparent Peer Review file

## Data Availability

The raw image test data generated in this study have been deposited in the Zenodo database under record number 21970445 with associated https://doi.org/10.5281/zenodo.21970444 [https://zenodo.org/records/21970445].

## Code availability

The source code used for offline processing and compositing of previously acquired HistoCAM image stream data is permanently archived on github as a publication-specific release at https://github.com/cooopermaira/HistoCAM_Nature_Communications.git. This repository contains the frozen version of the software associated with this study and is not the active development repository for HistoCAM. The software is made available under the Creative Commons Attribution-NonCommercial 4.0 International (CC BY-NC 4.0) license.The public software release contains the code required to process previously acquired HistoCAM datasets and generate the composite image representations described in this study. It does not include software for microscope control, camera acquisition, hardware synchronization, or operation of the full HistoCAM telemetry acquisition system. These components are specific to the instrumentation and hardware configuration described in this study and are not publicly distributed.

## References

[1] Evans, A. J. et al. Validating whole slide imaging systems for diagnostic purposes in pathology: Guideline Update From the College of American Pathologists in Collaboration With the American Society for Clinical Pathology and the Association for Pathology Informatics. *Arch. Pathol. Lab. Med.* **146**, 440–450 10.5858/arpa.2020-0723-CP (2022). 34003251

[2] Liu, Y. et al. Artificial intelligence–based breast cancer nodal metastasis detection: insights into the black box for pathologists. *Arch. Pathol. Lab. Med.* **143**, 859–868 10.5858/arpa.2018-0147-OA (2019). 30295070

[3] Capper, D. et al. DNA methylation-based classification of central nervous system tumours. *Nature* **555**, 469–474 10.1038/nature26000 (2018). PMC6093218 29539639

[4] Coudray, N. et al. Classification and mutation prediction from non–small cell lung cancer histopathology images using deep learning. *Nat. Med.* **24**, 1559–1567 10.1038/s41591-018-0177-5 (2018). PMC9847512 30224757

[5] Cruz-Roa, A. et al. Accurate and reproducible invasive breast cancer detection in whole-slide images: A Deep Learning approach for quantifying tumor extent. *Sci. Rep.* **7**, 46450 10.1038/srep46450 (2017). PMC5394452 28418027

[6] Ehteshami Bejnordi, B. et al. Diagnostic assessment of deep learning algorithms for detection of lymph node metastases in women with breast cancer. *JAMA* **318**, 2199–2210 10.1001/jama.2017.14585 (2017). PMC5820737 29234806

[7] Steiner, D. F. et al. Impact of deep learning assistance on the histopathologic review of lymph nodes for metastatic breast cancer. *Am. J. Surg. Pathol.* **42**, 1636–1646 10.1097/PAS.0000000000001151 (2018). PMC6257102 30312179

[8] Evans, A. J. et al. US Food and Drug Administration approval of whole slide imaging for primary diagnosis: a key milestone is reached and new questions are raised. *Arch. Pathol. Lab. Med.* **142**, 1383–1387 10.5858/arpa.2017-0496-CP (2018). 29708429

[9] Ardon, O. et al. Digital pathology operations at a tertiary cancer center: Infrastructure requirements and operational cost. *J. Pathol. Inform.* **14**, 100318 10.1016/j.jpi.2023.100318 (2023). PMC10550754 37811334

[10] Perincheri, S. et al. An independent assessment of an artificial intelligence system for prostate cancer detection shows strong diagnostic accuracy. *Mod. Pathol.* **34**, 1588–1595 10.1038/s41379-021-00794-x (2021). PMC8295034 33782551

[11] Chen, P.-H. C. et al. An augmented reality microscope with real-time artificial intelligence integration for cancer diagnosis. *Nat. Med.* **25**, 1453–1457 10.1038/s41591-019-0539-7 (2019). 31406351

[12] Chalfoun, J. et al. MIST: accurate and scalable microscopy image stitching tool with stage modeling and error minimization. *Sci. Rep.* 10.1038/s41598-017-04567-y (2017). PMC5504007 28694478

[13] Chow, S. K. et al. Automated microscopy system for mosaic acquisition and processing. *J. Microsc.* **222**, 76–84 10.1111/j.1365-2818.2006.01577.x (2006). 16774516

[14] Hsu, W.-Y., Poon, W.-F. P. & Sun, Y.-N. Automatic seamless mosaicing of microscopic images: enhancing appearance with colour degradation compensation and wavelet-based blending. *J. Microsc.* **231**, 408–418 10.1111/j.1365-2818.2008.02052.x (2008). 18754995

[15] Khurd, P. et al. Global error minimization in image mosaicing using graph connectivity and its applications in microscopy. *J. Pathol. Inform.* **2**, S8 10.4103/2153-3539.92039 (2011). PMC3312714 22811964

[16] Legesse, F. B. et al. Seamless stitching of tile scan microscope images. *J. Microsc.* **258**, 223–232 10.1111/jmi.12236 (2015). 25787148

[17] Preibisch, S., Saalfeld, S. & Tomancak, P. Globally optimal stitching of tiled 3D microscopic image acquisitions. *Bioinformatics* **25**, 1463–1465 10.1093/bioinformatics/btp184 (2009). PMC2682522 19346324

[18] Schroeder, J. L., Bakalar, M., Pohida, T. J. & Balaban, R. S. Rapid overlapping-volume acquisition and reconstruction (ROVAR): automated 3D tiling for high-resolution, large field-of-view optical microscopy. *J. Microsc.* **243**, 103–110 10.1111/j.1365-2818.2011.03490.x (2011). PMC3115409 21348869

[19] Sun, C., Beare, R., Hilsenstein, V. & Jackway, P. Mosaicing of microscope images with global geometric and radiometric corrections. *J. Microsc.* **224**, 158–165 10.1111/j.1365-2818.2006.01687.x (2006). 17204063

[20] Gherardi, A. & Bevilacqua, A. Manual stage acquisition and interactive display of digital slides in histopathology. *IEEE J. Biomed. Health Inform.* **18**, 1413–1422 10.1109/JBHI.2013.2291998 (2014). 25014942

[21] Pradhan, D. et al. Evaluation of panoramic digital images using Panoptiq for frozen section diagnosis. *J. Pathol. Inform.* **7**, 26 10.4103/2153-3539.181770 (2016). PMC4872477 27217976

[22] *iSTIX - VMscope* https://www.vmscope.com/product-overview/istix/ (2021).

[23] *Digitizing Slides Using a Manual Microscope and Digital Camera | Olympus LS* https://evidentscientific.com/en/applications/manual-whole-slide-imaging (2025).

[24] *Microscope Based-Digital Pathology System - Augmentiqs* https://www.augmentiqs.com/microscope-based-digital-pathology-system/ (2025).

[25] *Affordable Microscope Slide Scanner | Microvisioneer mvSlide* https://www.microvisioneer.com/mvslide (2025).

[26] Rublee, E., Rabaud, V., Konolige, K. & Bradski, G. ORB: An efficient alternative to SIFT or SURF. *Int. Conf. Comput. Vis.* 10.1109/ICCV.2011.6126544 (2011).

[27] Lowe, D. G. Distinctive Image Features from Scale-Invariant Keypoints. *Int. J. Comput. Vis.* **60**, 91–110 10.1023/B:VISI.0000029664.99615.94 (2004).

[28] Hou, W. & Wei, Y. Evaluating the resolution of conventional optical microscopes through point spread function measurement. *iScience* **26**, 107976 10.1016/j.isci.2023.107976 (2023). PMC10562796 37822495

[29] Björkman, M., Bergström, N. & Kragic, D. Detecting, segmenting and tracking unknown objects using multi-label MRF inference. *Comput. Vis. Image Underst.* **118**, 111–127 10.1016/j.cviu.2013.10.007 (2014).

[30] Hosseini, M. S. et al. Computational pathology: A survey review and the way forward. *J. Pathol. Inform.* **15**, 100357 10.1016/j.jpi.2023.100357 (2024). PMC10900832 38420608

[31] Gutman, D. A. et al. The digital slide archive: a software platform for management, integration and analysis of histology for cancer research. *Cancer Res.* **77**, e75–e78 10.1158/0008-5472.CAN-17-0629 (2017). PMC5898232 29092945

[32] Flach, R. N. et al. Significant inter- and intralaboratory variation in gleason grading of prostate cancer: a nationwide study of 35,258 patients in The Netherlands. *Cancers* **13**, 5378 10.3390/cancers13215378 (2021). PMC8582481 34771542

[33] Ravi, N. et al. SAM 2: Segment Anything in Images and Videos. *International Conference on Learning Representations* https://openreview.net/forum?id=Ha6RTeWMd0 (2025).

[34] Alfasly, S. et al. Rotation-Agnostic Image Representation Learning for Digital Pathology. *Proceedings of the IEEE/CVF Conference on Computer Vision and Pattern Recognition*, 11683–11693 10.1109/CVPR52733.2024.01110 (2024).

[35] Xu, H. et al. A whole-slide foundation model for digital pathology from real-world data. *Nature* **630**, 181–188 10.1038/s41586-024-07441-w (2024). PMC11153137 38778098

[36] Oquab, M. et al. DINOv2: Learning Robust Visual Features without Supervision. *Transactions on Machine Learning Research*, https://openreview.net/forum?id=a68SUt6zFt (2024).

[37] Neidlinger, P. et al. A deep learning framework for efficient pathology image analysis. *Nat. Commun.* **17**, 5740 10.1038/s41467-026-74918-9 (2026). PMC13324285 42386722

[38] Huang, Z. et al. A pathologist–AI collaboration framework for enhancing diagnostic accuracies and efficiencies. *Nat. Biomed. Eng.* **9**, 455–470 10.1038/s41551-024-01223-5 (2025). 38898173

[39] Brunyé, T. T., Nallamothu, B. K. & Elmore, J. G. Eye-tracking for assessing medical image interpretation: A pilot feasibility study comparing novice vs expert cardiologists. *Perspect. Med. Educ.* **8**, 65–73 10.1007/s40037-019-0505-6 (2019). PMC6468026 30977060

[40] Krupinski, E. A. Current perspectives in medical image perception. *Atten. Percept. Psychophys.* **72**, 1205–1217 10.3758/APP.72.5.1205 (2010). PMC3881280 20601701

[41] Kundel, H. L., Nodine, C. F. & Krupinski, E. A. Computer-displayed eye position as a visual aid to pulmonary nodule interpretation. *Invest Radio.* **25**, 890–896 10.1097/00004424-199008000-00004 (1990). 2394571

[42] Sultana, J., Qin, R. & Yin, Z. Seeing Through Expert’s Eyes: Leveraging Radiologist Eye Gaze and Speech Report with Graph Neural Networks for Chest X-Ray Image Classification. *ACCV 2024: 17th Asian Conf. Comput. Vis.* 10.1007/978-981-96-0901-7_9 (2024).

[43] Paalvast, O. et al. Radiology AI Lab: evaluation of radiology applications with clinical end-users. *J. Imaging Inform. Med.* **38**, 4303–4311 10.1007/s10278-025-01453-2 (2025). PMC12701144 40097768

[44] Ghezloo, F. et al. Robust ROI detection in whole slide images guided by pathologists’ viewing patterns. *J. Imaging Inf. Med*. **38**, 439–454 10.1007/s10278-024-01202-x (2025). PMC11811336 39122892

[45] Huang, W. et al. Act Like a Pathologist: Tissue-Aware Whole Slide Image Reasoning. *Proceedings of the IEEE/CVF Conference on Computer Vision and Pattern Recognition*, 6972–6981 (2026).

[46] Wang, S. et al. Pathology-CoT: Learning Visual Chain-of-Thought Agent from Expert Whole Slide Image Diagnosis Behavior. *ArXiv* abs/2510.04587 (2025). 10.1038/s41551-026-01739-y 42498734

[47] Chen, C. et al. Evidence-based diagnostic reasoning with multi-agent copilot for human pathology. ArXiv abs/2506.20964 (2025).

[48] Shannon, C. E. Communication in the presence of noise. *Proc. IRE* **37**, 10–21 (1949).

[49] Fischler, M. A. & Bolles, R. C. Random sample consensus: a paradigm for model fitting with applications to image analysis and automated cartography. *Commun. ACM* **24**, 381–395 10.1145/358669.358692 (1981).

[50] *Home - JUCE* https://juce.com/ (2025).

[51] OpenCV Library. *OpenCV v. 4.6.0.* OpenCV https://opencv.org/release/opencv-4-6-0/ (2022).

[52] Fixstars Corporation. *cuda-bundle-adjustment v. 2.1.1* GitHub https://github.com/fixstars/cuda-bundle-adjustment/releases/tag/2.1.1 (2024).

[53] Shaikovski, G. et al. PRISM: A Multi-Modal Generative Foundation Model for Slide-Level Histopathology. *arXiv:2405. 10254v2 [eess. IV]* 10.48550/arXiv.2405.10254 (2024).

