# Supplementary material for: Ambient, real-time digitization and datafication of glass slide microscopy towards AI-at-the-microscope
Source: Nat Commun. 2026 Sep 17;17:10933. doi: 10.1038/s41467-026-77887-1 (PMC13680511; doi:10.1038/s41467-026-77887-1)
Supplement: Supplementary file 1 [file 41467_2026_77887_MOESM10_ESM.pdf]

nature portfolio

# Peer Review File

# Ambient, real-time digitization and datafication of glass slide microscopy towards AI-at-the-microscope

Corresponding Author: Dr J. Quincy Brown

**This file contains all reviewer reports in order by version, followed by all author rebuttals in order by version.**

Version 0:

Reviewer comments:

Reviewer #2

(Remarks to the Author)
HistoCAM is a new hybrid analog–digital pathology platform that ambiently digitizes the entire glass-slide diagnostic session in real time—without changing how pathologists use their microscopes. The system attaches a large-format 31-megapixel, high-space-bandwidth camera to a standard trinocular microscope head and continuously streams full–field–of–view video as the pathologist reviews the slide. Custom software mosaics these frames into a multi-resolution slide image (MRSI) spanning 2× to 40× magnifications, while simultaneously capturing rich behavior telemetry such as magnification usage, search paths, and dwell times.

The authors propose that this may be an inflection point for computational pathology—integrating AI into the most common existing workflow (glass-slide microscopy) instead of requiring full laboratory digitization. Additionally, in terms of implications and significance, the authors claim that HistoCAM:
1. Dramatically lowers barriers to digital pathology adoption.
2. Enables AI where WSI scanners are not feasible or cost-effective.
3. Produces novel multimodal datasets linking images + behavior (telemetry).
4. Supports real-time quality assurance and expert-AI collaboration.
5. Represents a shift from digitization → datafication in pathology.

Although I see points 1 and 2, I can push back on those two claims. Looking at the workstation used in the study (CPU, GPUs, system RAM, and SSD); these components are necessary for HistoCAM compositing (stitching), along with tile embedding and AI inferencing tasks, which includes SAM2 ViT-L. Such a workstation is quite expensive (approximately $10-$14K in my estimate). As a single workstation, the capital cost is much lower than that of a whole-slide scanner. However, in a large practice of twenty-five pathologists, the total number of workstations can run close to a whole slide scanner. This also does not account for the deployment costs in these local workstation environments. Most pathologists lack the expert knowledge to deploy such AI models. Likewise, most IT teams and the cost of IT expertise to run these tools on pathologists' workstations in large practices are also expensive. I would like to see the author’s response.

Regarding points 3 through 5, I largely agree. Points 3 and 4 are feasible within current whole slide imaging (WSI) environments. However, for point 3 specifically, it is important to note that pathologists' viewing behavior in WSI systems—captured through telemetry—has not yet been systematically studied or compared to behavior in traditional microscope-based workflows. There is also a generational variable: younger pathologists who trained with digital tools may exhibit different viewing patterns than more experienced pathologists who transitioned from microscopes to WSI.

These questions present compelling opportunities for neuropsychology-style studies, similar to the eye-tracking research conducted in radiology by Dr. Elizabeth Krupinski. Incorporating a discussion of how telemetry has been leveraged in radiology and how similar approaches could strengthen pathology workflows would significantly enhance the paper's impact.

Furthermore, while the authors elegantly describe the technical aspects of HistoCAM that enable computational decision support at the microscope, the comparison provided in Extended Data Table 1 would benefit from a more explicit and expanded discussion. From this reviewer's perspective, this is a critical point to address, particularly because articles submitted to Nature Communications should meet a high bar of innovation and clearly demonstrate advancement over

existing technologies. To that end, it would be helpful for the authors to clearly articulate, point by point, what each microscope-based digitization system (Panoptiq, EasyStitch, Google ARM, and Augmentiqs) can and cannot do relative to HistoCAM. Highlighting how HistoCAM surpasses these systems across multiple dimensions—such as what is actually digitized, the degree of workflow disruption and field-of-view coverage, multi-resolution behavior, telemetry and "datafication," AI integration, and data size/efficiency—would substantially strengthen the manuscript.

Finally, I believe the authors should clarify whether the MRSI image file and the associated spatiotemporal telemetry/registration data are stored together or separately. I believe they are separate and modularized (but it is difficult to decipher). If these elements are indeed modularized, the manuscript should explicitly state this, as the distinction has important implications for both computational pathology and medico-legal documentation. Modularizing the raw image data from the telemetry, annotation, and registration layers reduces friction in computational workflows by allowing each component to be processed, versioned, and updated independently. This separation not only improves interoperability across analytic platforms but also minimizes downstream data-intensive bottlenecks when training or deploying AI models at scale. Equally important, modularization preserves an immutable, traceable archive of the original image while permitting flexible updates to metadata such as annotations, diagnoses, or consent restrictions—an increasingly critical requirement for medico-legal defensibility and auditability.

Making explicit whether the MRSI image and spatiotemporal telemetry data follow a modular architecture is important for readers to appreciate the system's strengths and limitations. Evidence across imaging science and AI methodology shows that modular file architectures—where raw images remain fixed while telemetry, annotations, and provenance metadata evolve as separate but linkable layers—enhance computational tractability, promote interoperability, and provide clearer medico-legal documentation trails. For these reasons, modularization represents not just a technical preference but a best practice increasingly endorsed across imaging, informatics, and AI research communities. Adding the author's commentary on this can strengthen the paper's argument that HistoCAM, as a platform, enables AI research/collaboration and datafication.

Reviewer #3

(Remarks to the Author)
The manuscript introduces HistoCAM, a hardware–software system mounted on a pathology microscope that captures images during manual slide examination. It records all user actions, tiles the scanned regions, accounts for magnification changes, and integrates the data into a single consolidated output.

Finally, the acquired data are processed to reconstruct the pathologist's navigation across the slide, and the system applies image processing and AI-based tools including segmentation and feature extraction, area measurements and more to assist in interpreting the scanned regions and support the pathologist work.

Recording the fields of view examined by an expert while eye-balling through the microscope is not a new concept, as noted in the introduction. However, the manuscript presents an improved approach that addresses several limitations of earlier systems and introduces enhanced capabilities that advance this line of work.

Nevertheless, it remains unclear whether this approach is well-suited for routine pathological diagnostics. First, the cost analysis omits one of the most substantial expenses: the extensive data-communication infrastructure, large-scale data-storage capacity, and high-performance computing resources required to operate such a system. In addition, the proposed workflow would require pathologists to perform two distinct diagnostic steps, one at the microscope and another at a computer workstation, resulting in a significantly increased workload compared with workflows that rely exclusively on traditional microscopy or exclusively on digital WSI-based diagnostics.

Moreover, the current approach would require purchasing a dedicated HistoCam unit for every workstation and pathologist, leading to substantially higher overall costs compared with acquiring a limited number of WSI systems that operate continuously (24/7) and can efficiently serve multiple users. In summary, although the concept is interesting, I do not believe it is suitable as a scalable or broadly applicable solution for pathology departments. It may be an interesting add-on for clinical research, training and alike. Having said that, I view this point as a contextual comment rather than a major concern, and it does not alter my overall assessment of the manuscript.

The manuscript is well written, technically detailed, and provides thorough explanations that cover all relevant aspects of the system's development. I have no doubt that it is a robust system that performs well for its purpose, as described. However, in my view, the manuscript is highly specialized and primarily focused on technical implementation. Its emphasis on engineering design and developmental narrative suggests that it would be more appropriately suited for a more technical journal in microscopy, optics, or engineering, rather than Nature Communications. Indeed, a substantial portion of the manuscript is dedicated to technical descriptions that, while important, are not aligned with the broader conceptual and scientific scope typically expected for this journal.

Other issues:
1. The example provided for determining the status of prostatectomies (lines 239-276) correctly illustrates a challenge where many slides must be examined. The authors present a useful software application that assists the pathologist in marking tumorous regions on the monitor. However, to use the system as proposed, the pathologist must first manually scan all slides under the microscope and only afterward perform the evaluation on the computer. In contrast, WSI systems eliminate this

redundancy by allowing the pathologist to begin the assessment directly on the monitor, using equivalent tools for annotation and tumor evaluation. Therefore, I think that this example highlights a practical disadvantage of the current approach rather than its benefit.

2. Line 135, define SIFT here (it is defined in line 164).
3. Few references are not fully quoted, for example, Reference 1 (missing volume and page numbers), references 36, 37 where the source is missing (ArXiv).

Version 1:

Reviewer comments:

Reviewer #2

(Remarks to the Author)
I see the comments of the other reviewer. The authors did address my comments thoroughly.

(Remarks on code availability)
I do not possess sufficient expertise to evaluate the code.

**Open Access** This Peer Review File is licensed under a Creative Commons Attribution 4.0 International License, which permits use, sharing, adaptation, distribution and reproduction in any medium or format, as long as you give appropriate credit to the original author(s) and the source, provide a link to the Creative Commons license, and indicate if changes were made.
In cases where reviewers are anonymous, credit should be given to 'Anonymous Referee' and the source.
The images or other third party material in this Peer Review File are included in the article’s Creative Commons license, unless indicated otherwise in a credit line to the material. If material is not included in the article’s Creative Commons license and your intended use is not permitted by statutory regulation or exceeds the permitted use, you will need to obtain permission directly from the copyright holder.
To view a copy of this license, visit https://creativecommons.org/licenses/by/4.0/

We thank the Reviewers for donating their time and expertise to a constructive review of our work. We believe the manuscript is improved with the edits suggested by the reviewers. Below is a point-by-point response to the reviewer critiques. Corresponding changes to the manuscript text are denoted by red text.

**Reviewer Comments:**

**Reviewer 2:**
**HistoCAM is a new hybrid analog–digital pathology platform that ambiently digitizes the entire glass-slide diagnostic session in real time—without changing how pathologists use their microscopes. The system attaches a large-format 31-megapixel, high-space-bandwidth camera to a standard trinocular microscope head and continuously streams full–field–of–view video as the pathologist reviews the slide. Custom software mosaics these frames into a multi-resolution slide image (MRSI) spanning 2× to 40× magnifications, while simultaneously capturing rich behavior telemetry such as magnification usage, search paths, and dwell times.**

**The authors propose that this may be an inflection point for computational pathology—integrating AI into the most common existing workflow (glass-slide microscopy) instead of requiring full laboratory digitization. Additionally, in terms of implications and significance, the authors claim that HistoCAM:**

**1. Dramatically lowers barriers to digital pathology adoption.**
**2. Enables AI where WSI scanners are not feasible or cost-effective.**
**3. Produces novel multimodal datasets linking images + behavior (telemetry).**
**4. Supports real-time quality assurance and expert-AI collaboration.**
**5. Represents a shift from digitization → datafication in pathology.**

**Although I see points 1 and 2, I can push back on those two claims. Looking at the workstation used in the study (CPU, GPUs, system RAM, and SSD); these components are necessary for HistoCAM compositing (stitching), along with tile embedding and AI inferencing tasks, which includes SAM2 ViT-L. Such a workstation is quite expensive (approximately $10-$14K in my estimate). As a single workstation, the capital cost is much lower than that of a whole-slide scanner. However, in a large practice of twenty-five pathologists, the total number of workstations can run close to a whole slide scanner. This also does not account for the deployment costs in these local workstation environments. Most pathologists lack the expert knowledge to deploy such AI models. Likewise, most IT teams and the cost of IT expertise to run these tools on pathologists' workstations in large practices are also expensive. I would like to see the author's response.**

We appreciate Reviewer 2's observations. We offer the following points in response:

1. The original custom workstation described in our manuscript was in some ways "overbuilt" because it was intended to be a robust and capable development platform. We built this workstation from parts for approximately $10,000 in 2023 dollars, but we acknowledge that buying a similar commodity workstation with the same specs would be more expensive in today's dollars. However, much of the work described in the manuscript was geared at improving computational efficiency to ease practical deployment of real-time capabilities. In addition, in ongoing efforts to reduce adoption barriers, while the manuscript was under review, we obtained one of the first-run of the NVIDIA DGX Spark

"personal AI supercomputers," which natively contains a GPU with the NVIDIA GB10 Blackwell architecture, 128 GB of unified CPU/GPU memory, as well as a 10 GigE ethernet connection suitable for running our camera system. We modified the HistoCAM software to integrate the Spark into the HistoCAM prototype. These "mini-AI-supercomputer" systems (the NVIDIA DGX Spark, Dell Pro Max AI, and similar) cost ~$4,000 USD, and are *dramatically* smaller in size: approximately 2 in × 6 in × 6 in and weighing only 3 pounds, while maintaining full performance in our pipeline. The transition to these unified memory computers offers benefits to HistoCAM in several ways. A 60% price reduction should substantially ease the cost burden on a per installed unit basis. In addition, with the GB10 Blackwell GPU architecture and unified CPU/GPU memory, we demonstrated that these computers are readily capable to perform real-time streaming and datafication, as well as deploy the AI assistance integrated within HistoCAM.

We have added additional sections to our manuscript (Results and Methods) describing the deployment of the NVIDIA DGX Spark and provide results demonstrating equivalent performance to the original dual-GPU workstation:

Results (starting on line 363):

"**Deployment on an edge AI computing system**

As previously described, one of the major barriers for the adoption of digital pathology is the substantial financial investment required to purchase, maintain, and operate high-throughput FDA-approved whole slide scanners. HistoCAM is intended to provide access to key benefits of digital pathology without requiring a whole-slide scanning system; however we recognize that the cost and footprint of HistoCAM hardware must also be minimized to promote broader adoption. To reduce costs as well as physical footprint, we deployed the HistoCAM system on a compact, relatively low-cost unified memory computer (NVIDIA DGX Spark). We verified that this system is capable of operating the HistoCAM hardware and supporting real-time image acquisition, compositing, and AI inference at substantially lower cost and physical footprint compared to our original custom workstation (**Supplementary Video 7**).

Specifically, the DGX Spark architecture integrates CPU and GPU resources within a shared memory space (128 GB unified system memory), which reduces data transfer overhead between the CPU and GPU processing units, and mitigates bottlenecks commonly encountered in CPU-GPU systems with discrete memory. By eliminating the need to copy image data between separate memory pools, the system enables efficient handling of large image datasets, and supports GPU-accelerated computation across the full memory capacity. In addition, the platform provides a native CUDA-enabled environment, facilitating integration of AI models and image analysis pipelines within the HistoCAM framework. Together, these characteristics support a compact and scalable computational implementation that may lower barriers to deployment in resource-constrained settings."

Methods (starting on line 530):

"Recently, we obtained an NVIDIA DGX Spark from the first production run. The NVIDIA DGX Spark is a compact, 2 in. × 6 in. × 6 in., 3 lb. personal AI computer incorporating a 20-core ARM Grace CPU and a Grace Blackwell architecture GB10 GPU with 5th Gen Tensor Cores optimized for AI tasks, all with 128 GB of LPDDR5X coherent unified system memory. Importantly, it features a 4TB NVMe SSD drive and integrated 10 GigE ethernet, meaning that it is capable of running the

HistoCAM camera hardware as well as handling the data bandwidth streaming from the camera. We modified the HistoCAM software to utilize the DGX Spark platform, and verified that we can obtain the same (or better) real-time performance in terms of data acquisition, compositing, and AI inferencing as compared to our home-built workstation platform previously described. Due to its performance, small size and low power consumption, and relatively low cost, this platform (which is also available from other vendors, such as the Dell Pro Max) is currently the preferred computer system for HistoCAM deployment."

2. Additionally, we would like to emphasize that although the startup cost for integrating HistoCAM into a large pathology practice may be comparable to that of purchasing a *single* whole slide scanner to service the practice, if the goal in is to digitize every slide that requires pathologist review in order to provide access to routine digital capabilities, then even modestly-sized institutions would need more than one slide scanner to cover the volume of slides generated. <u>In addition, they would also require</u> personnel to run the slide scanners and perform QC, since in general, pathologists are not responsible for slide scanning duties, which are usually handled by dedicated technicians. Using the Reviewer's scenario of a practice with 25 pathologists, each reviewing a modest slide load of only 100 slides per day, this would come out to 2,500 slides per day requiring digitization. The throughput of FDA-approved scanners for primary diagnosis range from 40-60 slides per hour, which gives an upper end of 480 slides scanned per 8-hour person-shift, or 1,440 slides per day if run continuously (three 8-hour consecutive person-shifts). Therefore, in order to keep up with the caseload while keeping turnaround time restricted to 1 day after slide generation, the practice would need 2 slide scanners and 3 full-time personnel, or 3 slide scanners and 2 FTE personnel, etc. etc. With HistoCAM, the only operator required is the pathologist themselves, since the digitized composite image is a byproduct of work that would already be completed by the pathologist.

3. Regarding the comment about pathologist knowledge and IT support, we acknowledge that routine use of HistoCAM would generate more data than a "non-digitized" practice would normally do. However, we note a few key points:
    a. The amount of data generated per slide is optimized compared to standard whole slide scanning, since the multi-resolution slide images are digitized in a tiered magnification approach and exactly reflecting the diagnostic process. Therefore the data required to archive HistoCAM slide images is a fraction of that required for 40X WSI images.
    b. It is not necessary that all HistoCAM data would need to be fully archived if IT is an issue, since the actual diagnostic act is still rendered using the microscope and the glass slide. Real-time use would allow for real-time actions *using the glass slide* based on the image data, and the system could be configured to only save the most important images and data for archiving. The slide could always be re-digitized later if needed.
    c. For deployment of AI tools, the integration of the AI models is handled within config files after initial installation (which can easily be packaged with the HistoCAM software). The pathologist would only need minimal training to become familiar with the AI-assisted annotation tools which have been designed for ease of use. As for the use of AI-assisted classification and ease of adoption by pathologists, we believe that the HistoCAM platform could be a compelling platform to improve the pathologist-AI integration loop, which is the subject of ongoing and future work.

**Regarding points 3 through 5, I largely agree. Points 3 and 4 are feasible within current whole slide imaging (WSI) environments. However, for point 3 specifically, it is important to note that**

**pathologists' viewing behavior in WSI systems—captured through telemetry—has not yet been systematically studied or compared to behavior in traditional microscope-based workflows. There is also a generational variable: younger pathologists who trained with digital tools may exhibit different viewing patterns than more experienced pathologists who transitioned from microscopes to WSI.**

**These questions present compelling opportunities for neuropsychology-style studies, similar to the eye-tracking research conducted in radiology by Dr. Elizabeth Krupinski. Incorporating a discussion of how telemetry has been leveraged in radiology and how similar approaches could strengthen pathology workflows would significantly enhance the paper's impact.**

We thank the Reviewer for the comment. We wholeheartedly agree with these points, and we are also greatly intrigued by the possibilities of studying pathologist viewing behavior between glass slide and digital whole slide reviews. Our work has indeed been influenced by Dr. Elizabeth Krupinski's pioneering research in visual perception and diagnostic behavior, both in radiology[1] and pathology,[2] and we are also inspired by related work from Dr. Joanne Elmore and colleagues.[3-6] User-tracking telemetry has been extensively studied in radiology to understand how radiologists search images, interact with viewing systems, and make diagnostic decisions. The most widely used approach is eye-tracking, which records fixation locations, scan paths, and dwell times to characterize visual search behavior during image interpretation. These studies have shown that diagnostic errors often arise from search or recognition failures and that expert radiologists exhibit more efficient gaze patterns than trainees.[1,7,8] Eye-tracking data has also been incorporated into machine-learning pipelines to guide model attention toward clinically relevant image regions (e.g., gaze-guided CNNs for chest radiographs).[9] More recently, integrated telemetry frameworks have been proposed that combine interaction logs, gaze data, and contextual workflow signals to objectively measure how AI tools influence radiologist behavior and decision-making during clinical image interpretation.[10]

Experience from radiology suggests several promising research directions for digital pathology. Indeed, Krupinski et al. conducted some of the early work in user-tracking for digital pathology,[2] even prior to FDA approval of whole-slide imaging for primary diagnosis. First, telemetry could enable learning of diagnostic search trajectories, in which models are trained from pathologist navigation traces to predict which regions of a slide should be examined following the initial low-magnification overview.[6] Second, telemetry could support workflow-aware evaluation of AI systems, allowing investigators to quantify how AI assistance affects diagnostic efficiency, search patterns, and time-to-diagnosis.[10] Third, user-interaction data could serve as a form of weak supervision for AI training, generating attention maps or evidence paths based on signals of attention, saliency, and even intent derived from telemetry features such as magnification changes, navigation paths, and dwell times. Such telemetry signals can currently be derived from digital whole-slide image review (e.g., screen viewport tracking and eye-tracking) and are also available in the HistoCAM approach described here.

However, as shown in **Supplementary Fig. 4**, **Supplementary Fig. 5**, and **Supplementary Video 3**, viewing patterns differ substantially between glass-slide microscopy and digital slide review, even for the same pathologist evaluating the same slide. Because the user interface for digital slide viewing – keyboard, mouse, and monitor – is the same interface used for navigating the broader computer environment, accidental user events (e.g., clicks or key presses) can introduce noisy signals. As a result, it may be more difficult to infer user intent from digital interaction logs than from microscope workflows, which require

more deliberate physical movements (e.g., changing objective lenses, physically translating the slide) that more clearly reflect attention or saliency. Ultimately, however, both modalities provide rich telemetry data that could be used to model the stepwise diagnostic reasoning used by pathologists, potentially enabling a new generation of human-aligned computational pathology systems. Recent advances in this area for digital slide interpretation are particularly exciting,[11-14] and could be further informed by the unique human-microscope-slide interaction telemetry datasets generated by HistoCAM, which are the subject of ongoing and future work

**To address the Reviewer's feedback**, we have added the following discussion of these points to our Discussion, and we agree with the reviewer that introducing these points, especially given the recent momentum in the field towards human-aligned computational pathology systems, will magnify impact of the paper:

Discussion, starting on line 436:

"User-tracking telemetry has been extensively studied in radiology to understand how radiologists search images, interact with viewing systems, and make diagnostic decisions. The most widely used approach is eye-tracking, which records fixation locations, scan paths, and dwell times to characterize visual search behavior during image interpretation. These studies have shown that diagnostic errors often arise from search or recognition failures and that expert radiologists exhibit more efficient gaze patterns than trainees.[1,7,8] Eye-tracking data has also been incorporated into machine-learning pipelines to guide model attention toward clinically relevant image regions.[9] More recently, telemetry frameworks combining gaze data, interaction logs, and workflow context have been proposed to measure how AI systems influence radiologist behavior and decision-making.[10]

These findings suggest several potential roles for telemetry in digital pathology. Navigation traces could be used to learn diagnostic search trajectories, enabling models to predict which regions of a slide should be examined after the initial low-magnification overview.[6] Telemetry may also enable workflow-aware evaluation of AI systems, quantifying how AI assistance alters search patterns, efficiency, and time-to-diagnosis.[10] Finally, interaction data may provide a form of weak supervision for AI training, generating attention maps or evidence paths based on signals such as magnification changes, navigation paths, and dwell times. Such signals can be derived from digital slide viewers through viewport tracking or eye-tracking. However, as shown in **Supplementary Figures 4 and 5** and **Supplementary Video 3**, viewing behavior differs substantially between glass-slide microscopy (obtained *via* HistoCAM telemetry) and digital slide review (obtained *via* viewport tracking), even for the same pathologist reviewing the same slide on the microscope vs. digitally. Because digital WSI slide viewers rely on general-purpose input devices (i.e. keyboard, mouse), interaction logs may include noisy signals from incidental user actions, whereas HistoCAM microscope navigation requires more deliberate movements (i.e. objective lens changes) that may more directly reflect diagnostic intent. Nevertheless, both modalities provide rich telemetry data that may enable modeling of pathologists' stepwise diagnostic reasoning and support the development of human-aligned computational pathology systems, an area of growing recent interest.[11-14]"

**Furthermore, while the authors elegantly describe the technical aspects of HistoCAM that enable computational decision support at the microscope, the comparison provided in Extended Data Table 1 would benefit from a more explicit and expanded discussion. From this reviewer's perspective, this is a critical point to address, particularly because articles submitted to Nature**

**Communications should meet a high bar of innovation and clearly demonstrate advancement over existing technologies. To that end, it would be helpful for the authors to clearly articulate, point by point, what each microscope-based digitization system (Panoptiq, EasyStitch, Google ARM, and Augmentiqs) can and cannot do relative to HistoCAM. Highlighting how HistoCAM surpasses these systems across multiple dimensions—such as what is actually digitized, the degree of workflow disruption and field-of-view coverage, multi-resolution behavior, telemetry and "datafication," AI integration, and data size/efficiency—would substantially strengthen the manuscript.**

Thank you for this input. Following is a point-by-point comparison of HistoCAM to the competing solutions. As in our Extended Data Table 1, we group the solutions into 3 types: 1) AR overlay systems inside the microscope body (Google ARM, Augmentiqs), 2) "Manual" video-mosaicking add-ons (Panoptiq, Motic EasyStitch), and 3) Ambient comprehensive multi-resolution digitization / datafication + AI (HistoCAM).

1) Regarding the **AR overlay systems**, Google's ARM boasts impressive SBTP at over twice the rate of HistoCAM, while also offering full coverage of the pathologist FOV. However, the system is limited by its larger pixel size giving a lower spatial sampling ability than HistoCAM. Therefore, the ratio between digital and optical sampling resolution (a measure of how well the imaging system samples the optical information available to the pathologist by the microscope eyepieces) is 16% lower than HistoCAM. Most importantly, the ARM acts only as a real-time assistant for exactly where the pathologist FOV is, since it relies on a physical overlay of AI results over the current pathologist FOV using projectors and mirrors inside the microscope. Augmentiqs (a similar approach to ARM) offers the highest spatial sampling with the smallest pixel size of the group, but a relatively low SBTP at 359.31 MHz. Their small sensor size diagonal of 15.78 mm leads to only a 30% FOV coverage of the 2x objective, which leaves 70% of the pathologist FOV excluded from any digital record at this lowest magnification, which is usually used for an "overview" of the entire slide. Generally, these AR overlay systems do not offer 1) capture of the pathologist's telemetry, 2) archival storage of the data collected, nor 3) reconstruction of the whole slide image reviewed by the pathologist. Therefore, no digital composites are created for any secondary use-cases, nor for application of computational assists to the entire slide image.

2) Regarding the **manual video-mosaicking add-ons**, current product offerings have pathologists perform a manual scan while observing the live feed from the onboard camera on a computer monitor to ensure coverage. The main limitations of these systems currently is their limited FOV coverage, with ViewsIQ's Panoptiq and Motic's EasyStitch Pro covering only 18.37% and 15.7% of the pathologist FOV respectively. Panoptiq offers registration of isolated high-mag fields-of-view within the constructed mosaics, but not a full "ambient" multi-resolution slide image of the full diagnostic review. As a result of the underfilled FOV, these systems require the pathologist to divert their attention from the microscope to the computer monitor as they translate the slide, to ensure complete coverage. This causes a major disruption to the clinical workflow of the pathologist, as, in order to avoid gaps in the digital image, they are required to monitor progress of image stitching on the computer screen instead of analyzing the microscope slide through the oculars. Therefore, these solutions preclude any capture or analysis of standard diagnostic microscope slide-viewing behavior.

3) In contrast, **ambient comprehensive multi-resolution slide digitization / datafication + AI** using HistoCAM addresses these shortcomings, while also maintaining the highest SBP, second highest SBTP, and highest ratio of digital-to-optical SBP sampling (i.e., HistoCAM covers 98% of the

available optical SBP at low magnifications, and 100% at higher magnifications, which is unmatched by the other techniques). These combined hardware capabilities required significant algorithmic and software developments to handle the unprecedented stream of high-speed, high-resolution streaming image data into real-time image composites and computational assists. HistoCAM's large format camera sensor allows for 98.78% FOV coverage and 98% coverage of the optical SBP at 2x magnification (and 100% optical SBP coverage at higher magnifications), meaning it is better matched for the optics than either of the other two solutions, while offering nearly the same amount of FOV coverage at the lowest magnification as the best AR overlay solutions. Critically, it captures an adequately-sampled multi-resolution digital image of everything viewed by the pathologist (best digitization approach), enables direct computational assist and AI application on the full slide image (most comprehensive computation / AI image substrates), as well as collects rich telemetry data, while not interfering with the established analog workflow of pathologists (only datafication approach). These features clearly differentiate the feature-set and capabilities of HistoCAM from the competing solutions.

To address the Reviewer's comment, we have made the following modifications to the paper. We added a Supplementary Note, referring to our original Extended Data Table 1:

**"Supplementary Note 1: Comparison of microscope-based digitization approaches**

To further clarify the distinction between HistoCAM and prior microscope-based digitization systems (Extended Data Table 1), we group existing approaches into three categories: (i) augmented reality (AR) heads-up display (HUD) overlay systems (e.g., Google ARM, Augmentiqs), (ii) manual video-mosaicking systems (e.g., Panoptiq, EasyStitch), and (iii) ambient multi-resolution digitization (HistoCAM).

AR overlay systems provide real-time AI assistance within the microscope field of view but are fundamentally limited to analysis of the current field of view, since the heads-up-display (HUD) relies on physical projection of light from a screen onto the eye through the microscope eyepiece(s). These systems do not generate a persistent, multi-resolution digital representation of the slide, nor do they reconstruct the pathologist's full diagnostic trajectory. As a result, they do not support whole-slide-level digital representation and computation, archival reconstruction of the diagnostic review, or capture of diagnostic telemetry beyond the instantaneous field of view.

Manual video-mosaicking systems enable reconstruction of larger slide regions but rely on user-directed scanning with visual feedback on a monitor to ensure coverage. Because these systems substantially underfill the microscope field of view (**Extended Data Table 1**), they require pathologists to divert attention away from the eyepieces, disrupting the standard diagnostic workflow. In addition, these approaches typically operate at a single magnification at a time and do not capture the multi-resolution, spatiotemporal nature of diagnostic slide review, limiting their ability to represent natural search behavior.

In contrast, HistoCAM performs ambient, full-field-of-view digitization during routine microscope use, capturing nearly the entire optical field of view without requiring changes to pathologist behavior (**Extended Data Table 1**). This enables reconstruction of a multi-resolution slide image (MRSI) that reflects the regions actually examined, while simultaneously recording detailed telemetry of magnification changes, navigation paths, and dwell times. By combining comprehensive field-of-view coverage, multi-scale integration, and real-time AI compatibility, HistoCAM uniquely enables both workflow-aligned

digitization and datafication of the diagnostic process, distinguishing it from prior AR-based and mosaicking approaches."

We also revised the following sentences in the Results section (starting at line 71):

"A detailed comparison of HistoCAM with prior microscope-based digitization and augmentation systems, highlighting differences in field-of-view and optical resolution coverage, workflow integration, multi-resolution behavior, and telemetry capture, is provided in **Extended Data Table 1** and **Supplementary Note 1**. In contrast to these competing methods, we "ambiently" generated digital images of the entire specimen that were directly reflective of natural glass slide diagnostic processes by leveraging a high space-bandwidth-time-product camera capable of fully digitizing the optical information available to the pathologist through the eyepieces, and a computationally-efficient processing approach. These distinctions reflect a shift from frame-based or manually mediated digitization approaches toward an ambient, task-driven paradigm aligned with natural diagnostic behavior."

**Finally, I believe the authors should clarify whether the MRSI image file and the associated spatiotemporal telemetry/registration data are stored together or separately. I believe they are separate and modularized (but it is difficult to decipher). If these elements are indeed modularized, the manuscript should explicitly state this, as the distinction has important implications for both computational pathology and medico-legal documentation. Modularizing the raw image data from the telemetry, annotation, and registration layers reduces friction in computational workflows by allowing each component to be processed, versioned, and updated independently. This separation not only improves interoperability across analytic platforms but also minimizes downstream data-intensive bottlenecks when training or deploying AI models at scale. Equally important, modularization preserves an immutable, traceable archive of the original image while permitting flexible updates to metadata such as annotations, diagnoses, or consent restrictions—an increasingly critical requirement for medico-legal defensibility and auditability.**

**Making explicit whether the MRSI image and spatiotemporal telemetry data follow a modular architecture is important for readers to appreciate the system's strengths and limitations. Evidence across imaging science and AI methodology shows that modular file architectures—where raw images remain fixed while telemetry, annotations, and provenance metadata evolve as separate but linkable layers—enhance computational tractability, promote interoperability, and provide clearer medico-legal documentation trails. For these reasons, modularization represents not just a technical preference but a best practice increasingly endorsed across imaging, informatics, and AI research communities. Adding the author's commentary on this can strengthen the paper's argument that HistoCAM, as a platform, enables AI research/collaboration and datafication.**

We thank the reviewer for highlighting the importance of clarifying the data architecture underlying the HistoCAM system outputs. The reviewer is correct in the assumption that the MRSI image data, and the associated spatiotemporal telemetry, annotations, labels, and AI inference outputs, are stored in a modular architecture rather than as a single monolithic file. Specifically, the reconstructed MRSI data are stored as the primary (stable) image layer; while telemetry, annotation, segmentation, and model output information – including magnification level, spatial navigation trajectories, temporal dwell information registration metadata, user annotations, segmentation output masks, etc. – are stored as separate but linked metadata layers. These layers are connected through shared spatial and temporal coordinates, allowing

telemetry and user interaction data to be mapped back to the reconstructed image, while preserving independence between the components. We agree with the reviewer that this is the preferred approach, for all of the reasons that the reviewer mentioned. To respond to this comment, we have inserted the following text at the end of the "Results: Multi-Resolution Slide Images Generated in Real Time" section (starting at line 219):

"The reconstructed MRSI data and the associated spatiotemporal telemetry are stored using a modular architecture. The composited image pyramid serves as the primary (stable) image layer, while telemetry data, including frame timestamps, magnification level, spatial registration coordinates, and navigation trajectories, are stored as linked metadata layers referenced through shared spatial and temporal indices. This structure allows the image content, telemetry data, and derived metadata to be processed and versioned independently while preserving an immutable visual record of the captured microscopy session. Such modularization facilitates interoperability with downstream computational pathology pipelines and enables flexible integration of additional metadata layers – such as user annotations, segmentation masks, AI outputs, or provenance information – without modification of the underlying image data."

**Reviewer 3:**
**The manuscript introduces HistoCAM, a hardware–software system mounted on a pathology microscope that captures images during manual slide examination. It records all user actions, tiles the scanned regions, accounts for magnification changes, and integrates the data into a single consolidated output. Finally, the acquired data are processed to reconstruct the pathologist's navigation across the slide, and the system applies image processing and AI-based tools including segmentation and feature extraction, area measurements and more to assist in interpreting the scanned regions and support the pathologist work.**

**Recording the fields of view examined by an expert while eye-balling through the microscope is not a new concept, as noted in the introduction. However, the manuscript presents an improved approach that addresses several limitations of earlier systems and introduces enhanced capabilities that advance this line of work. Nevertheless, it remains unclear whether this approach is well-suited for routine pathological diagnostics. First, the cost analysis omits one of the most substantial expenses: the extensive data-communication infrastructure, large-scale data-storage capacity, and high-performance computing resources required to operate such a system.**

We appreciate the Reviewer's assessment of our work. Regarding the cost analysis, the first Reviewer had a similar concern. We refer to the points made in that discussion above. In addition, we point out our recent work to implement the system on a compact NVIDIA DGX Spark mini-supercomputer, which costs about the same as a well-equipped personal laptop. In that regard, the relatively modest hardware costs of a single HistoCAM add-on to an existing microscope – approximately ~$14,000 retail including the camera (~$7,000), trinocular microscope beamsplitter head (if not already available) (~$2,500), and an NVIDIA DGX Spark or Dell Pro Max AI computer ($4,000) – are about 20-25X lower than the cost of a single high-throughput slide scanner ($200,000 - $300,000 +). Therefore, outfitting 25 pathologists with HistoCAM systems (including computers for each) would cost about the same as a single high-throughput WSI scanner. This is not to mention the fact that HistoCAM does not require technicians, since images are generated during standard clinical glass slide review.

We do acknowledge that adoption of HistoCAM by institutions that have not made the transition to digital pathology would result in much more image data generation that could stress IT infrastructure, if the desire is to archive all data. However, we make 2 points in response.

1) HistoCAM can be useful in real-time without permanent image archiving. Note that in contrast to WSI, the actual diagnosis is still performed on the glass slide using the microscope, and *the digital image is not required to render the diagnosis*. In this case, HistoCAM is available as a real-time assist, but the individual user and institution can decide whether and how much of the data need to be retained archivally (for instance, institutions may only keep certain image or annotation outputs as an adjunct to pathology reports, etc). ***Therefore, HistoCAM provides a flexible alternative that creates access to digital infrastructure and assists during real-time glass slide review, without necessarily tying institutions to the data burden*** (since the diagnostic act is performed on the glass slide, not the digital image).

2) Even if the institution decides to archive all HistoCAM data, in comparison to standard high-resolution WSI, in which the entire slide is digitized at 40X for primary diagnosis, in our case, only the magnification needed to render the diagnosis is used, and therefore *we find that HistoCAM MRSI file sizes are up to 2 orders of magnitude smaller than corresponding WSI images* – therefore reducing the necessary IT requirements.

**We have modified the manuscript to add our recent work using the low-cost NVIDIA DGX Spark computing system (see response to Reviewer 1).** We hope our response above, and these manuscript edits, clarifies the potential for routine HistoCAM adoption as compared to current approaches with regards to the required computational and data-storage infrastructure.

**In addition, the proposed workflow would require pathologists to perform two distinct diagnostic steps, one at the microscope and another at a computer workstation, resulting in a significantly increased workload compared with workflows that rely exclusively on traditional microscopy or exclusively on digital WSI-based diagnostics. In summary, although the concept is interesting, I do not believe it is suitable as a scalable or broadly applicable solution for pathology departments. It may be an interesting add-on for clinical research, training and alike. Having said that, I view this point as a contextual comment rather than a major concern, and it does not alter my overall assessment of the manuscript.**

We thank the reviewer for the comment, and are grateful that this is not a major concern. To respond, we sincerely apologize if our presentation of the HistoCAM concept was unclear. The proposed workflow does not require two distinct steps. The diagnosis of the slide occurs using the microscope, just as in the traditional workflow. HistoCAM simply serves as a "digital sidekick" which digitizes this gold standard process in real-time, without disruption to the existing workflow.

(A useful analogy is driver assists in modern cars. Drivers control the car, while sensors collect data and process it in real-time to assist the driver.)

Likewise, simultaneously as the slide is reviewed, HistoCAM provides immediate access to digital data, unlocking otherwise unavailable digital assists. The microscope is connected to the computer workstation, and the two are used in concert, at the same time. As such, HistoCAM is not 2 steps, but 1 efficient, hybrid step, and therefore does not multiply workload.

**The manuscript is well written, technically detailed, and provides thorough explanations that cover all relevant aspects of the system's development. I have no doubt that it is a robust system that performs well for its purpose, as described. However, in my view, the manuscript is highly specialized and primarily focused on technical implementation. Its emphasis on engineering design and developmental narrative suggests that it would be more appropriately suited for a more technical journal in microscopy, optics, or engineering, rather than Nature Communications. Indeed, a substantial portion of the manuscript is dedicated to technical descriptions that, while important, are not aligned with the broader conceptual and scientific scope typically expected for this journal.**

**Other issues:**
**1. The example provided for determining the status of prostatectomies (lines 239-276) correctly illustrates a challenge where many slides must be examined. The authors present a useful software application that assists the pathologist in marking tumorous regions on the monitor. However, to use the system as proposed, the pathologist must first manually scan all slides under the microscope and only afterward perform the evaluation on the computer. In contrast, WSI systems eliminate this redundancy by allowing the pathologist to begin the assessment directly on the monitor, using equivalent tools for annotation and tumor evaluation. Therefore, I think that this example highlights a practical disadvantage of the current approach rather than its benefit.**

We greatly appreciate the feedback from the Reviewer. We would like to re-emphasize that HistoCAM is positioned as an adoptable bridge to digitization for institutions that have not adopted routine slide digitization, which inarguably is the vast majority of institutions. If one starts from the premise that slides are already digitized, then the digital tools available in the HistoCAM software and the ones available in WSI digital slide image management systems look very similar. However, here we make 3 key points:

1. The pathologist does not "manually scan" the slides – they just perform their standard diagnostic workup using the microscope, and digitization happens as a by-product. Diagnosis happens in one step using the glass slide. As described in further detail below, the software tools then provide a highly advantageous workflow uplift for quantification and documentation tasks, and can inform the glass slide review (the "driver assist").
2. HistoCAM could bring a lower-barrier, digital workflow uplift to <u>institutions that have not or cannot make the full shift to routine digital whole slide scanning</u>, as outlined in detail in the manuscript.
3. HistoCAM provides unique telemetry data that could have independent value, which are simply not available with WSI scanners.

We will here focus on points 1 and 2 as they seem to be more relevant to the Reviewer's comment. If the pathologist does not have access to a routine digital slide scanning infrastructure (this is currently the majority of pathologists), then viewing the slides at the microscope is the current diagnostic platform. Traditionally, when working up a case such as a radical prostatectomy or other surgical excisions of various organs, various pathological features must be addressed in the pathology report, such as location and extent of close/positive margin status, volume and location of tumor, tumor grade, presence of lymphovascular / seminal vesicle / perineural invasion, mitotic figures, and so on. It is routine practice for pathologists to **<u>physically</u>** mark on the slide the presence and location of certain features with an ink marker, as well as take manual notes as they progress through the slides, which are addressed and synthesized into the report at the conclusion of the slide review. The pathologist will then go back through

their annotations on the slides and their notes to confirm whether they have seen histological features which must be remarked upon in the pathology report. It is important to note this secondary annotation and synthesis process is already occurring during manual slide review – it is currently just manual and "analog."

HistoCAM is meant to assist in this process, by providing immediate access to digital images. As a slide is reviewed, images are immediately composited and made available. The pathologist does not need to divert their attention from the microscope during the review, as all of the data is collected and composited in real-time, and the unique hardware architecture of the platform ensures that nothing the pathologist sees is missed. However, as each slide is reviewed, pathologists can then turn their attention to their monitor, and *digitally* annotate, segment, label, and measure all salient features of the diagnosis. These are all stored digitally and can be easily recalled and summarized into the final pathology report, along with digital images or screenshots corresponding to features of interest, as well as quantitative measurements (instead of "eyeballing" items such as tumor volume, distance of tumor from the margin, length of margin involvement, specific locations of each pathologic feature, etc.). In this sense, HistoCAM is a practical advantage over the current way of using the glass slide microscope, which is the more apt comparison (rather than comparing against routine digitization of WSI which is not widely available). Given that the routine adoption of WSI has been extremely slow and recent industrial and market assessments do not predict rapid uptake of routine WSI in the near future, our platform could offer a more practical short-term alternative bridge to digitization.

In addition, WSI, even when available, is not used in certain scenarios (such as on-site frozen section analysis). HistoCAM could provide digitization and workflow uplift in those scenarios, generating data that would have otherwise been unavailable.

**2. Line 135, define SIFT here (it is defined in line 164).**

Thank you for this suggestion, we have made the following change:

**"**We opted to use ORB for single-resolution feature matching as it is computationally more efficient than Scale Invariant Feature Transform (SIFT[15]) while offering similarly accurate feature extraction and matching across images of the same magnification level."

**3. Few references are not fully quoted, for example, Reference 1 (missing volume and page numbers), references 36, 37 where the source is missing (ArXiv).**

We thank you for catching these issues and have corrected the references.


1 Krupinski, E. A. Current perspectives in medical image perception. *Atten Percept Psychophys* **72**, 1205-1217 (2010). https://doi.org/10.3758/APP.72.5.1205

2 Krupinski, E. A., Graham, A. R. & Weinstein, R. S. Characterizing the development of visual search expertise in pathology residents viewing whole slide images. *Hum Pathol* **44**, 357-364 (2013). https://doi.org/10.1016/j.humpath.2012.05.024

3 Kerr, K. F. *et al.* Effects of Prior Diagnosis on Second Opinions and Pathologist Viewing Behaviors: Results from a Randomized Trial in Breast Pathology. *Med Decis Making* **46**, 76-87 (2026). https://doi.org/10.1177/0272989X251368886

4 Brunye, T. T., Mitroff, S. R. & Elmore, J. G. Artificial intelligence and computer-aided diagnosis in diagnostic decisions: 5 questions for medical informatics and human-computer interface research. *J Am Med Inform Assoc* **33**, 543-550 (2026). https://doi.org/10.1093/jamia/ocaf123

5 Lotter, W. *et al.* Influence of Mammography Acquisition Parameters on AI and Radiologist Interpretive Performance. *Radiol Artif Intell* **7**, e240861 (2025). https://doi.org/10.1148/ryai.240861

6 Ghezloo, F. *et al.* Robust ROI Detection in Whole Slide Images Guided by Pathologists' Viewing Patterns. *J Imaging Inform Med* **38**, 439-454 (2025). https://doi.org/10.1007/s10278-024-01202-x

7 Brunyé, T. T., Nallamothu, B. K. & Elmore, J. G. Eye-tracking for assessing medical image interpretation: A pilot feasibility study comparing novice vs expert cardiologists. *Perspectives on Medical Education* **8**, 65-73 (2019). https://doi.org/10.1007/s40037-019-0505-6

8 Kundel, H. L., Nodine, C. F. & Krupinski, E. A. Computer-displayed eye position as a visual aid to pulmonary nodule interpretation. *Invest Radiol* **25**, 890-896 (1990). https://doi.org/10.1097/00004424-199008000-00004

9 Sultana, J., Qin, R. & Yin, Z. 142-158 (Springer Nature Singapore).

10 Paalvast, O. *et al.* Radiology AI Lab: Evaluation of Radiology Applications with Clinical End-Users. *Journal of Imaging Informatics in Medicine* **38**, 4303-4311 (2025). https://doi.org/10.1007/s10278-025-01453-2

11 Huang, W. *et al.*

12 Wang, S. *et al.* Pathology-CoT: Learning Visual Chain-of-Thought Agent from Expert Whole Slide Image Diagnosis Behavior. *ArXiv* **abs/2510.04587** (2025).

13 Neidlinger, P. *et al.* A deep learning framework for efficient pathology image analysis. *ArXiv* **abs/2502.13027** (2025).

14 Chen, C. *et al.* Evidence-based diagnostic reasoning with multi-agent copilot for human pathology. *ArXiv* **abs/2506.20964** (2025).

15 Lowe, D. G. Distinctive Image Features from Scale-Invariant Keypoints. *International Journal of Computer Vision* **60**, 91–110 (2004). https://doi.org/10.1023/B:VISI.0000029664.99615.94

We thank the reviewers for donating their time and expertise to review of our manuscript.  We respond to the reviewer comments below.

**Reviewer #2 (Remarks to the Author):**

**I see the comments of the other reviewer. The authors did address my comments thoroughly.**

Thank you for your re-review of our manuscript.

**Reviewer #2 (Remarks on code availability):**

**I do not possess sufficient expertise to evaluate the code.**
