# Supplementary material for: Ambient, real-time digitization and datafication of glass slide microscopy towards AI-at-the-microscope
Source: Nat Commun. 2026 Sep 17;17:10933. doi: 10.1038/s41467-026-77887-1 (PMC13680511; doi:10.1038/s41467-026-77887-1)
Supplement: Supplementary file 2 [file 41467_2026_77887_MOESM2_ESM.pdf]

Description of Additional Supplementary Files

Supplementary Movie 1: Evaluation of slide coverage using HistoCAM v0 with limited camera field of view (related to Supplementary Fig. 1).

Supplementary Movie 2: Real-time multi-magnification image acquisition and compositing using HistoCAM v1.

Supplementary Movie 3: Comparison of pathologist search behavior between glass (HistoCAM) and digital workflows.

Supplementary Movie 4: Real-time tile-level inference for acid-fast bacteria detection.

Supplementary Movie 5: Real-time slide-level inference for Gleason grading.

Supplementary Movie 6: Rapid annotation and tile-level dataset generation using SAM2 integration.

Supplementary Movie 7: Real-time HistoCAM operation on a compact edge AI computing system.
