# Supplementary material for: Ambient, real-time digitization and datafication of glass slide microscopy towards AI-at-the-microscope
Source: Nat Commun. 2026 Sep 17;17:10933. doi: 10.1038/s41467-026-77887-1 (PMC13680511; doi:10.1038/s41467-026-77887-1)
Supplement: Supplementary file 3 [file 41467_2026_77887_MOESM1_ESM.docx]

**Supplementary Information for:**

**Ambient, real-time datafication and digitization of glass slide microscopy towards AI-at-the-microscope**

Cooper Maira,^1,*^ Max S. Cooper,^2,*^ Kimberly L. Ashman,^2,*^ Andrew B. Sholl,^3^ Sharon E. Fox,^4^ Shams Halat,^5^ David Manthey,^6^ Roni Choudhury,^6^ Jonathan Sears,^1^ Carola Wenk,^1^ J. Quincy Brown,^2, †,‡^ Brian Summa^1, †,‡^

*^1^Department of Computer Science, Tulane University, New Orleans, LA, USA*

*^2^Department of Biomedical Engineering, Tulane University, New Orleans, LA, USA*

*^3^Touro Infirmary, New Orleans, LA, USA*

*^4^Southeast Louisiana Veterans Healthcare System, New Orleans, LA, USA*

*^5^Department of Pathology and Laboratory Medicine, Tulane University, New Orleans, LA, USA*

*^6^Kitware, Inc., Clifton Park, NY, USA*

*^*^*These authors contributed equally

^†^These authors jointly supervised this work

^‡^***Corresponding authors:*** *J. Quincy Brown (*[*jqbrown@tulane.edu*](mailto:jqbrown@tulane.edu)*), Brian Summa (*[*bsumma@tulane.edu*](mailto:bsumma@tulane.edu)*)*

**Supplementary Note 1: Comparison of microscope-based digitization approaches**

To further clarify the distinction between HistoCAM and prior microscope-based digitization systems (Supplementary Table 1), we group existing approaches into three categories: (i) augmented reality (AR) heads-up display (HUD) overlay systems (e.g., Google ARM, Augmentiqs), (ii) manual video-mosaicking systems (e.g., Panoptiq, EasyStitch), and (iii) ambient multi-resolution digitization (HistoCAM).

AR overlay systems provide real-time AI assistance within the microscope field of view but are fundamentally limited to analysis of the current field of view, since the heads-up-display (HUD) relies on physical projection of light from a screen onto the eye through the microscope eyepiece(s). These systems do not generate a persistent, multi-resolution digital representation of the slide, nor do they reconstruct the pathologist’s full diagnostic trajectory. As a result, they do not support whole-slide-level digital representation and computation, archival reconstruction of the diagnostic review, or capture of diagnostic telemetry beyond the instantaneous field of view.

Manual video-mosaicking systems enable reconstruction of larger slide regions but rely on user-directed scanning with visual feedback on a monitor to ensure coverage. Because these systems substantially underfill the microscope field of view (Supplementary Table 1), they require pathologists to divert attention away from the eyepieces, disrupting the standard diagnostic workflow. In addition, these approaches typically operate at a single magnification at a time and do not capture the multi-resolution, spatiotemporal nature of diagnostic slide review, limiting their ability to represent natural search behavior.

In contrast, HistoCAM performs ambient, full-field-of-view digitization during routine microscope use, capturing nearly the entire optical field of view without requiring changes to pathologist behavior (Supplementary Table 1). This enables reconstruction of a multi-resolution slide image (MRSI) that reflects the regions actually examined, while simultaneously recording detailed telemetry of magnification changes, navigation paths, and dwell times. By combining comprehensive field-of-view coverage, multi-scale integration, and real-time AI compatibility, HistoCAM uniquely enables both workflow-aligned digitization and datafication of the diagnostic process, distinguishing it from prior AR-based and mosaicking approaches.

**Supplementary Table 1.** **Comparison of HistoCAM with other microscope-based digitization solutions.** Full field of view (FOV) coverage is only part of the equation, the other is whether the pixel size is sufficient to capture the available optical resolution. Therefore, we introduce the ratio of the digital to the optical space-bandwidth product (SBP) and space-bandwidth-time product (SBTP) at the lowest magnification to compare systems on their ability to cover the full FOV with sufficient sampling resolution.

**Supplementary Figure 1.** **Maximum measured stage translation velocity and corresponding frame rate as a function of camera sensor width to maintain coverage at maximum stage velocity.** A) Using a small-format high-frame rate camera (FLIR Blackfly S, 200 FPS) and video-mosaicking to calculate the translation between frames, we measured the speed of the stage as the pathologist reviewed specimens at each magnification level (red for 4X, yellow for 10X, green for 20X). The maximum stage translation speed was approximately 7 mm/s. B) Required frame rate as a function of camera sensor width for all objective lenses (blue for 2X, orange for 4X, grey for 10X, yellow for 20X, light blue for 40X, green for 60X, dark blue for 100X) to ensure gap-free coverage at maximum stage velocity. The Sony IMX342 sensor (FLIR Oryx 31 MP) selected for the HistoCAM prototype has a width of 22.3 mm, and the minimum frame rate to ensure overlap using all objective lenses (2X to 100X) was approximately 7 FPS, well below the maximum 26 FPS available with this camera.

**Supplementary Table 2.** **Comparison of system resolution, FOV, and space-bandwidth-product coverage between purely analog (i.e. visual observation through the eyepiece) and the HistoCAM digital capture, for a range of objective lenses tested in this work.**

**Supplementary Figure 2.** **The HistoCAM App user interface.** a) Streaming tool. Selecting this allows users to either (b) start a new recording stream from the camera or (c) re-stream an older recording from disk. d) Annotation tool. e) Distance measuring tool. f) Polygon annotation tool. g) AI-assisted segmentation tool (SAM2). Additional context-aware functions (such as deployment of pre-configured classification models, and labeling and logging features) are demonstrated in the supplementary videos.

**Supplementary Figure 3.** **Multi-resolution slide images (MRSIs) produced from microscope review.** MRSIs generated from multiscale pathologist slide reviews without altering the diagnostic behavior of the pathologist. Resolution maps showing objective magnification levels used by the pathologist at the microscope. The green overlays indicate the objective magnification (2X to 20X) used by the pathologist across the specimen area. Darker green overlays indicate higher objective magnification.

**Supplementary Figure 4.** **Telemetry data sets based on visual analytics of the pathologist at the microscope, demonstrating incomplete review.** a) Image data collected during the glass slide review are assembled into a single slide image. In addition, spatiotemporal data is collected, which comprise maps of b) utilized resolution (green), c) dwell time per pixel (red for high dwell time, blue for low dwell time), and the path of analysis (magenta to start, and cyan to end), both d) globally, and e) per lens used. Note the area of the slide missed in (a) by the initial slide review.

**Supplementary Figure 5. Re-review of a slide prompted by digital review (work check) after an initial HistoCAM microscope session.** a) The pathologist rater reviewed the slide initially using HistoCAM (session 1). b) After the MRSI was constructed (objective magnifications in green), c) the digital image was reviewed using a custom version of HistomicsUI that enables user behavior tracking similar to the HistoCAM process. An area of interest was identified digitally, in which nucleoli were not visible due to the use of 10X magnification in this area. The pathologist used this information to then return to the slide(digital zoom color coded as light blue for 0.125X, yellow for 0.25X, light purple for 0.5X, red for 1X, and dark blue for 2X), d) and review this area again using HistoCAM (session 2) but using the 20X objective lens (dark green). New frames from session 2 were integrated into the original MRSI, providing a final composite that reflected the full cumulative review. The nucleoli were then clearly visible in the updated MRSI.

**Supplementary Figure 6: HistoCAM annotation tools update current analog workflows.** 5.1: A digital image is taken showcasing the current method for tumor volume annotation in a given radical prostatectomy case. The pathologist used a fine point marker to draw dots (blue) around the tumor volume that was observed through the microscope, then estimated the total tumor volume for the case based on marked slides throughout the case. 5.2: HistoCAM is shown to offer convenient annotation in the same areas the pathologist would annotate tumor volume, while also giving the area of the tumor volume calculated in mm^2^. 5.2.A: The same slide from the image in 5.1 was scanned at low resolution using HistoCAM. 5.2.B: HistoCAM’s point-and-click polygon annotation (red) was used to annotate and automatically calculate the tumor area (in this case, 65 mm^2^). 5.2.C: The same polygon annotation tool was used to annotate the entirety of the tissue section area (in this case, 128 mm^2^). This process would quickly determine that the tumor for this slide represents approximately 65/128 = 50.78% of the total tissue area. 5.2.D-E shows a similar process of low magnification scanning and annotation of relevant tumor volumes on the slide. Note that in this case the slides were ink marked using the traditional process, but the use of HistoCAM would eliminate the need to do this manual marking, providing easy annotation, quantification, and digital archiving. Created using BioRender. Brown, Q. (2026) [<https://BioRender.com/fte69xh>]

**Supplementary Figure 7. Magnification usage and path of analysis by task elucidated by HistoCAM data.** The pathologist interaction pattern was mapped to the whole slide image of a kidney biopsy specimen. For this kidney biopsy specimen, the pathologist scanned the slide at 4x magnification (red). The pathologist only needed 10x (yellow) magnification to determine the tumor extent and 20x magnification (green) to grade nuclear features. This demonstrates how HistoCAM-derived search paths can be delineated by task – in this case, we asked the pathologist reviewer to indicate which task was being performed during the diagnosis of a kidney biopsy, which we used to encode the task-specific behavior into the rendered search paths. Such data could be used to provide automated labeling based on telemetry measures, or to prioritize areas of the slide for segmentation or classification based on the same.

**Supplementary Figure 8. Use of HistoCAM v0 to evaluate slide coverage when the pathologist looks solely through the eyepieces.** (a) Individual mosaics for each magnification level (red for 4X, blue for 10X, green for 20X) were recorded by a pathologist using HistoCAM v0, which was fitted with a small FOV camera that did not cover the entire eyepiece FOV. Note the gaps between the mosaics, which was due to the fact that the camera underfilled the visual information available to the pathologist. Therefore, they were unaware that gaps would be created, which demonstrates the need for cameras that fully cover the eyepiece FOV to accurately and ambiently reconstruct whole slide images reflective of the gold standard glass slide review process, without requiring users to monitor a computer screen to avoid gaps in coverage. (b) Individual sub-mosaics were registered to a subsequent WSI digital slide scan. (c) The centroids of each camera frame were convolved with the eyepiece FOV size for each objective lens to determine the full eyepiece FOV coverage, which was visualized as an overlay on the WSI. This demonstrated the potential for a large-FOV, large-format camera to adequately capture the entirety of the slide based on normal microscope analysis from the pathologist.

**Supplementary Figure 9. WSI with navigation path from pathologist review, using HistoCAM v0 frames convolved with the eyepiece FOV.** Red lines entail navigation with the 4X objective, yellow for the 10X objective, and green for the 20X objective. Navigation was performed on a FLIR Blackfly S 3 MP camera which was not capable of covering the entirety of the eyepiece FOV. These images showed proof of principle that the pathologist does in fact cover the entirety of the slide while reviewing glass slides under the microscope, indicating that if a camera with full FOV coverage was used, that complete digital slide coverage would be obtained. These data were used to design the HistoCAM v1 hardware.

**Supplementary Figure 10. A continuation of the data shown in Supplementary Figure 9.**

**Supplementary Figure 11. Comparison of user search behavior using glass slides at the microscope (HistoCAM, left) and standard digital whole slide images (collected using a custom implementation of HistomicsUI which allows viewport tracking during WSI review).** Elapsed time is shown on a gradient starting at 0% (cyan) and ending at 100% of elapsed time (magenta).

**Supplementary Figure 12. Heatmap showing total dwell time for each pixel and search path lines between glass (HistoCAM) and digital (WSI, HistomicsUI) user review.** White path lines indicate slower viewport movement while grey lines indicate faster movement. The white circles indicate the start of the analysis and open circles indicate the end. Areas of high dwell time are highlighted in red with areas of lower dwell time shown as a gradient to blue.
